# A three-dimensional scanning trapped-ion probe

**DOI:** 10.1126/sciadv.aec0794

**Published:** 2026-06-19

**Authors:** Tobias Sägesser, Shreyans Jain, Pavel Hrmo, Alexander Ferk, Matteo Simoni, Yingying Cui, Carmelo Mordini, Daniel Kienzler, Jonathan Home

**Affiliations:** ^1^ Department of Physics, ETH Zürich, 8093 Zürich, Switzerland.; ^2^ Quantum Center, ETH Zürich, 8093 Zürich, Switzerland.

## Abstract

The performance of a wide range of physical systems suffers from undesired fields from nearby surfaces, placing a premium on developing methods for understanding these imperfections. Trapped ions excel at sensing surface electric fields, but spatial scanning was limited to linear translations in previous work using radio-frequency traps, restricting the conclusions that could be drawn. Here, we demonstrate a single ion sensor confined using only static fields, consequently allowing three-dimensional position scanning and the possibility to isolate the trap from external noise. We measure static and time-varying electric and magnetic fields at distances between 50 and 450 micrometers from the metallic trap surface over a 200 micrometer–by–200 micrometer area, which we use to map charge distributions and surface noise, as well as to comprehensively discriminate between different noise sources. This approach opens up the potential to study a wide variety of materials, surface constitutions, and geometries, expanding the tool set of surface science.

## Teaser

An ion trapped by static fields senses electromagnetic disturbances in 3D, exposing noise sources troubling many quantum systems.

## INTRODUCTION

Fluctuations of electric fields at the surfaces of materials limit our ability to probe fundamental physics as well as the operation of a number of platforms for quantum information processing (*1*). In the context of sensing for Casimir (*2*, *3*) or gravitational forces (*4*, *5*), the resulting noise limits sensitivity, while quantum devices ranging from trapped ions (*6*, *7*) and Rydberg atoms (*8*, *9*) to superconducting circuits (*10*) and spin defects (*11*) find limits in coherence due to these effects. As a result, operation far from such surfaces has become common in multiple platforms, restricting a number of functions ranging from the use of microscopic traps to optical interfaces (*12*) and transduction (*13*). The cause of these fluctuations is not well understood because of the difficulty of separating out the interplay of the involved materials and their precise morphology (*14*, *15*) and crystal structure (*16*). For surfaces bordering on vacuum, it is also challenging to keep these surfaces clear of contaminants (*17*, *18*).

As a consequence of their sensitivity, these quantum systems have been used as sensors of the surrounding materials in an attempt to understand the sources of decoherence at play. Such sensing provides attractive prospects to further the understanding of various fabrication methods and materials (*19*, *20*), surface treatments (*21*–*23*), passivation techniques (*14*, *24*) and protective layers (*25*–*27*). However, conclusive results have been difficult to obtain for a number of reasons, including manufacturing and sample preparation variability as well as a difficulty in distinguishing noise of technical origin from surface noise (*28*, *29*).

No candidate system so far combines all properties of an ideal sensing platform. Atomic vapors (*30*), ensembles of spin defects (*31*, *32*), certain solid-state devices (*33*), and nanomechanical devices (*34*) are highly sensitive but lack in reconfigurability. Superconducting quantum interference devices can be deployed on scanning probes but are only susceptible to magnetic fields and trade-off resolution for sensitivity (*35*). Scanning-probe techniques with single solid-state spins such as nitrogen-vacancy centers even allow subnanometer-scale resolution (*35*) and can access both magnetic and electric fields. However, the achievable sensitivities are limited by the short coherence times of the spins due to the nearby surface (*14*) and are on the order of 100 nT/$\sqrt{\text{Hz}}$ and 26 kV/m/$\sqrt{\text{Hz}}$ (*35*, *36*). Both values are several orders of magnitude worse than what is achievable using a single trapped ion.

Trapped ions are highly sensitive to both electric and magnetic fields and are capable of placement with sub-micrometer resolution (*37*, *38*). Ions offer a high degree of control over both the motional and electronic quantum states, enabling advanced quantum sensing techniques. Magnetic fields have been measured with sensitivities of 4.6 pT/$\sqrt{\text{Hz}}$ (*39*) using Ramsey techniques, while the highest sensitivity to ac electric fields is reached in this work at 10 nitrogen-vacancy (nV)/m/$\sqrt{\text{Hz}}$. Ions further allow sensitivities beyond classical limits by harnessing entanglement (*40*), offering excellent prospects for precision sensing (*41*, *42*). These properties make trapped ions an excellent candidate platform for investigating noise above surfaces (*7*). However, implementations to date have lacked arbitrary positioning capability due to the use of radio frequency (rf)–confining fields, which only allow one-dimensional (1D) translations (*43*) and preclude the use as a true scanning probe. High sensitivity to noise originating in external equipment is a further detriment and has made it difficult to separate out technical sources from the fields caused by the surface (*7*). The use of high-voltage rf drives also raised the question whether observed behaviors are intrinsic to the materials or result from the drive (*44*). This calls for alternative solutions with enough flexibility for differentiating between these different sources and for performing 3D scanning.

Here, we present a single-ion sensor that addresses these deficiencies through the use of a microfabricated Penning trap (*45*). Confinement is achieved solely with static electric and magnetic fields, which enables arbitrary translations of the ion above the trap surface, removes doubts connected to the influence of rf-driving fields, and allows the trap electrodes to be temporarily isolated from external equipment. We demonstrate the 3D positioning control enabled by our platform by scanning the ion across a 200 μm–by–200 μm area of the metallic trap surface and at distances between 50 and 450 μm. We sample static and fluctuating electric and magnetic fields throughout this 3D volume using both classical techniques as well as quantum sensing protocols. The 3D sampling of the noise combined with the possibility to electrically isolate the trap allows us to extract the surface noise component from the measurements and, furthermore, to identify all additional sources of observed noise. The resulting data provide tests of models of electric-field noise above surfaces as well as insights into mechanisms of surface charging.

## RESULTS

### Penning microtrap

**Fig. 1.**
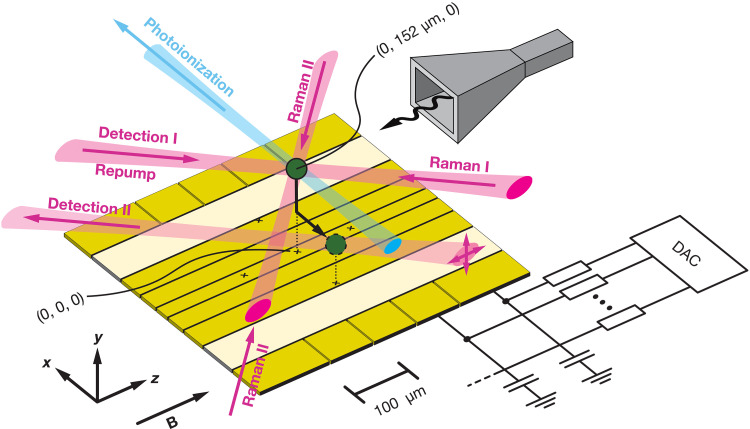
Trap chip and control fields. Schematic view of the central region of the trap, including an indication of the coordinate system and its origin. A typical transport path of an ion from the initial location at r = (0, 152 μm, 0) to a target location is shown. The propagation path of the laser beams used to cool, repump, and detect the ion is drawn, as well as the Raman beams used for ground-state cooling and coherent manipulation. The path of the PI laser is shown, as well as the microwave horn emitting radiation close to 83.2 GHz (not to scale). A schematic view of the DACs producing the slowly varying trapping voltages is added, together with the low-pass filters placed close to the trap. The weak oscillating drive used for coupling the radial modes during Doppler cooling and detection is applied to the outermost strip electrodes, indicated in beige.

The experimental apparatus consists of a microfabricated surface-electrode trap chip within a cryogenic vacuum apparatus, described in detail in earlier work (*46*). Figure 1 depicts the central region of the trap, together with illustrations of the wiring and voltage sources, as well as the laser beams and the microwave source used to control an ion. The trap chip consists of 25 gold electrodes deposited on a sapphire substrate, placed within a B=3 T static magnetic field oriented along z and held at a temperature of 6.5 K. Suitable voltages applied to all electrodes by a set of digital-to-analog converters (DAC) approximate an electric potential

$$\phi \left(r\right)=m\omega_{z}^{2}\left[2{\left(z-z_{0}\right)}^{2}-{\left(x-x_{0}\right)}^{2}-{\left(y-y_{0}\right)}^{2}\right]/\left(4e\right)$$ (1)

with a field null at a position $r_{0}=\left(x_{0},y_{0},z_{0}\right)$ at which we trap a single ${}^{9}{\text{Be}}^{+}$ ion. The confining curvature in the z direction results in an axial motion at frequency $\omega_{z}$, while the ion is confined in the radial *x*-*y* plane by the magnetic field. The combined electric and magnetic fields give rise to motion in the radial plane at the magnetron and modified cyclotron frequencies

$$\omega_{\pm }=\omega_{c}/2\pm \sqrt{\left(\omega_{c}^{2}-2\omega_{z}^{2}\right)}/2$$ (2)

where $\omega_{c}=\mathrm{eB}/m$ is the bare cyclotron frequency.

We use two sublevels of the 2s ^2^S_1/2_ ground-state manifold as an electronic spin qubit, with a frequency splitting of 83.2 GHz. Doppler cooling and fluorescence detection are performed using a closed-cycle transition between the upper qubit level and a sublevel of the 2p ^2^P_3/2_ manifold, at a wavelength close to 313 nm. During both operations, a weak mode-coupling drive oscillating at $\omega_{c}$ is applied to two electrodes (see Fig. 1), resulting in a coupling of the radial modes and thus enabling their simultaneous cooling (*46*). A repump laser is used to optically pump the ion into the bright qubit state. Two lasers with a wavelength close to 313 nm are phase-locked at a frequency difference matching the qubit splitting and can effect coherent Raman transitions between the qubit levels (*46*). These are also used for ground-state cooling of the ion motion when combined with the repump laser. Last, a photoionization (PI) laser with a wavelength close to 235 nm is used to ionize neutral Be atoms during inital loading. Further detail on the apparatus can be found in Supplementary text.

### Measurements of static electric fields

We first characterize the static electric fields present in our trap across a 3D grid of positions. Stray surface charging, caused for example by the interaction of laser beams with the trap, is a ubiquitous phenomenon in surface-electrode traps (*47*, *48*). The method involves transporting the ion to a desired location and subsequently recording its position as well as the shape of the point spread function on a camera. If electric fields beyond those included in our trap model are present, varying the curvature of the applied potential ϕ results in a shift of the observed ion position and a distortion of its shape. To calibrate these additional fields, we iteratively apply correction fields until both the shift and distortion are minimized.

**Fig. 2.**
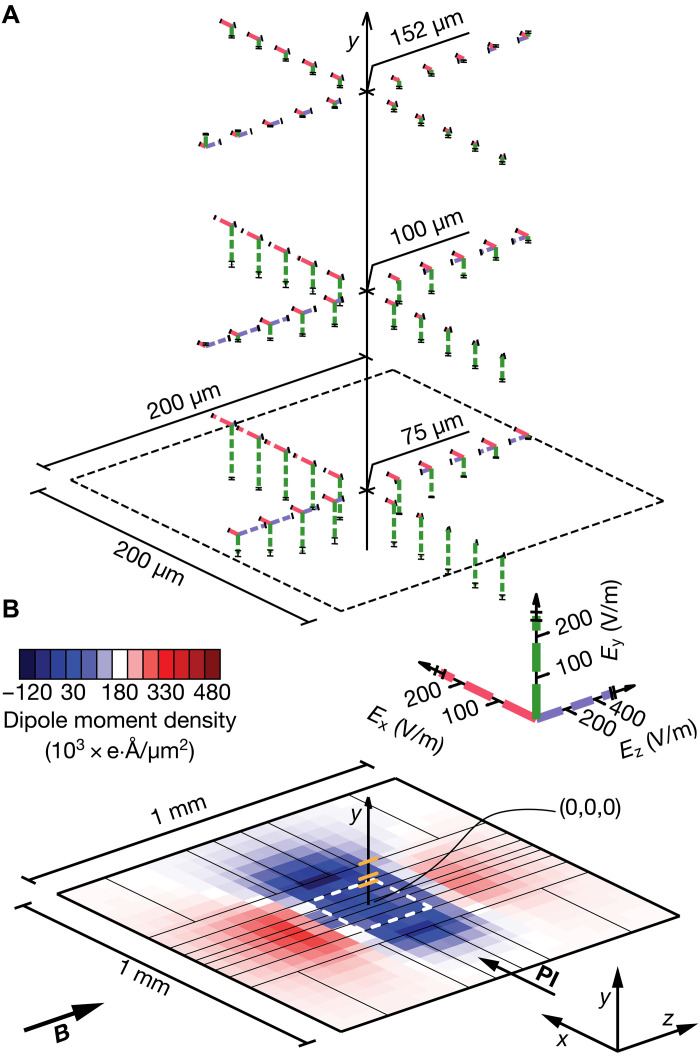
Measuring static electric fields. (**A**) Stray electric fields measured in 3D above a 200 μm–by–200 μm area. The three spatial components of the fields are drawn as shown in the magnified scale indication, with one dash corresponding to 100 V m^−1^ (200 V m^−1^) in the *x* and *y* (*z*) directions. Error bars are as detailed in Supplementary text. At a given ion-electrode distance (not to scale), measurement locations are spaced by 20 μm. (**B**) Inferred distribution of the dipole-moment density on a 1 mm–by–1 mm surface area, overlaid over the trap electrode layout. Note that the white color corresponds to 180 × 10^3^ *e* Ǻ μm^−2^. The white dashed rectangle indicates the measurement region, while the yellow dashes show the three ion heights to scale. The coordinate system, its origin, the magnetic field, and the path of the PI laser are indicated.

Figure 2A shows the stray electric fields measured above the chip surface in this way. We achieve accuracies in the range of 1 to 20 V m^−1^, with the measurement being most precise for fields along *x*, while out-of-plane fields are determined least accurately. This magnitude is comparable to the fields of tens of elementary charges at a distance of 100 μm. The sensitivity of this method improves with low ion mass, high imaging magnification, small camera pixels, and weak confinement.

Assuming that the observed fields are generated by charges on the trap surface, we use a model consisting of electric dipoles, which could be imagined to arise from photo-induced charges interacting with the surface (*47*). Contact potentials between the gold surface and adsorbed contaminants also result in dipolar fields (*49*). The electron distribution at the surface is distorted by the adsorbates, in particular, if the involved species differ in electronegativity (*19*). We infer the dipole distribution that yields the best fit to the measured fields and present the result in Fig. 2B. In the region where the 235-nm PI laser passes over the trap along *x*, a strip of negative dipoles is observed, which is in line with previously observed laser-induced charging (*47*). Because of the constraints of the vacuum apparatus, in particular, the number of optical paths to the trap, the PI laser can only traverse the trap along this direction, making additional tests of its effect difficult. It is however notable that the charging effect appeared to be largely saturated for the duration of data taking of about 6 months as continued operation of the setup only resulted in small changes to the observed stray fields (see Supplementary text and fig. S3). The data are best explained when allowing a uniform background dipole moment density *D* of 180 × 10^3^ *e* Å μm^−2^. We speculate that surface dipoles due to deposited beryllium atoms or other contaminants may be the cause. Converting *D* to a difference in work function $\Delta \phi =-\mathrm{eD}/\epsilon_{0}$ yields Δϕ≈−0.33 eV, compatible with the difference between beryllium and gold but also with submonolayer hydrocarbon contamination (*22*).

Similar visualization of electrical potentials is enabled at the atomic scale by scanning-probe sensors using NV centers (*50*) or quantum dots (*51*) but is restricted to working distances on the order of nanometers and measurement precision on the order of 1 × 10^6^ V m^−1^ (*52*). Penning microtraps as presented here enable the extension of the submicrometer spatial resolution typical for ion traps (*38*, *53*) to 3D and achieve a measurement precision of a few volts per meter as well as displacement across macroscopic ranges, enabling the study of charge distributions of 2D or 3D structures with micrometer-scale extents (*36*, *54*).

### Sensing electric field noise

Besides being affected by static fields, trapped ions also exhibit enhanced sensitivity to fluctuating electric fields at frequencies resonant with the ion motion. Such electric fields excite the oscillatory motion, which can be measured by coupling it to the electronic states using suitable laser or microwave fields before detecting the latter using state-dependent fluorescence (*55*). A long-standing challenge for surface science is that measured noise spectral densities above metallic electrodes are generally orders of magnitude higher than expected from Johnson noise (*6*), even after surface cleaning (*20*). There has been much effort to reveal the source of such noise (*7*, *44*), with candidate models including fluctuating patch potentials (*56*), dielectric layers (*57*, *58*), surface contaminants and adatoms (*17*, *59*, *60*), as well as two-level fluctuators with unknown physical realization (*61*). However, no single model fits the vast body of data, which is complicated by a large number of combinations of surface materials, temperatures, fabrication methods, geometries, and ion species. The electric-field noise spectral density $S_{E}$ is typically assumed (*7*) to show power-law scaling with the angular frequency ω, the distance to the surface *d* and the surface temperature *T*

$$S_{E}\propto \omega^{-\alpha }d^{-\beta }T^{\gamma }$$ (3)

Models predict specific values for some or all of the scaling exponents α, β, and γ. Aside from the ability to vary multiple of these parameters as well as the location in the plane above the surface, the Penning trap measurements reported here are notable for the absence of high-voltage rf-trapping fields and for the ability to detach all trap electrodes from the voltage sources during measurements, removing external noise present in the out-of-vacuum wiring (*46*).

To measure electric-field noise, an ion is first prepared in the ground state of the motional mode of interest λ and then transported to a target location for a variable wait time. A sideband probe pulse and subsequent fluorescence detection determine the average phonon number ${\bar{n}}_{\lambda }$ in the mode (*6*). A linear fit to the phonon number as a function of the wait time yields the heating rate ${\dot{\bar{n}}}_{\lambda }$. Regarding electric-field noise resonant with a motional frequency as a small perturbation compared to the trapping fields, a heating rate can be related to the power spectral density of the noise along the direction through

$$S_{E,z}\left(\omega_{z}\right)=4\hbar m\omega_{z}{\dot{\bar{n}}}_{z}/e^{2}$$ (4)

for the axial mode and through

$$S_{E,r}\left(\omega_{\pm }\right)=4\hbar m\left(\omega_{+}-\omega_{-}\right){\dot{\bar{n}}}_{\pm }/e^{2}$$ (5)

for the radial modes (*7*).

**Fig. 3.**
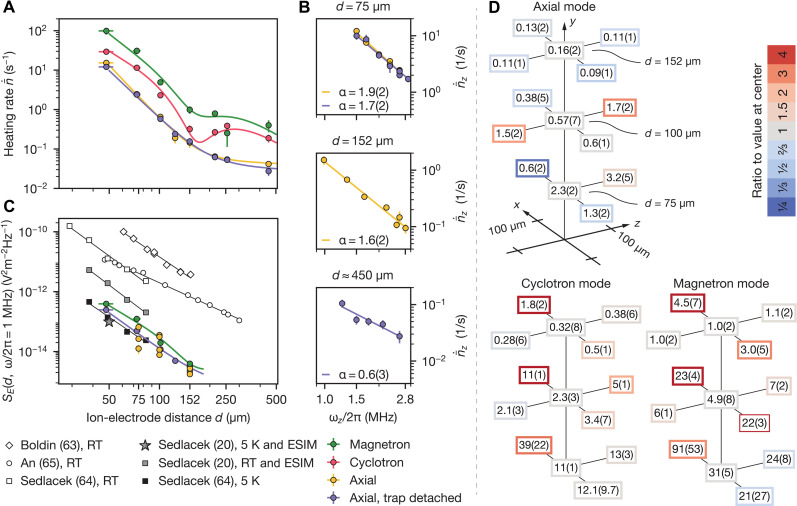
Sensing of electric-field noise. (**A**) Heating rates above the trap center, measured at distance *d*. Solid lines are fits as explained in the main text. Mode frequencies are $\omega_{z}=2\pi \times 2.6$ MHz, $\omega_{-}=2\pi \times 0.845$ MHz, and $\omega_{+}=2\pi \times 4.32$ MHz, with the radial modes chosen to avoid noise at discrete frequencies (see Supplementary text and fig. S5). Axial data are taken with and without the trap electrodes detached. Independent verification of *d* is detailed in Supplementary text and fig. S1. (**B**) Frequency scaling of the axial heating rates at three distances. Solid lines are power-law fits, yielding the exponent α. (**C**) Spectral noise densities inferred from the heating rates and rescaled to ω=2π×1 MHz using α=1.7. Data where surface noise likely dominates are shown, including from (D). Previous investigations with variable distance are added for comparison, rescaled using the reported exponents α. Data taken with traps at room temperature (RT) are shown with open symbols, while cryogenic (5 K) traps are marked with closed symbols. Gray symbols show data taken after ex situ ion milling (ESIM). (**D**) Heating rates in 3D, stated in phonons per second. Border colors indicate the ratio to the value at the respective central position. Mode frequencies are the same as in (A). All error bars correspond to 1 SD.

We investigate the scaling of the noise with the ion-electrode distance *d* and make a set of measurements at eight different heights above the center of the trap. As expected from work in rf traps, the data presented in Fig. 3A show increased noise affecting all modes as the ion is moved closer to the surface. At ion-electrode distances below 152 μm, this increase broadly takes a power-law form while the dependence on the height is less strong at larger separations. We explain these observations by assuming that the noise stems from several sources, which may differ in their position dependence. One component is assumed to arise from surface effects with power-law scaling $d^{-\beta }$ as per Eq. 3, identified with noise from microscopic processes on the trap surface. In addition, we assume the presence of noise due to thermal fluctuations in resistive elements of the trap circuitry as well as noise from technical equipment external to the apparatus. We find the strengths of the various contributions that explain the data best (see Supplementary text and fig. S6), leading to the fits shown in Fig. 3A.

Inspecting the data measured using the axial mode, surface noise is the dominant source below heights of d=152 μm, with the extracted power-law exponent being β=4.0±0.2. This result is consistent with β=4, as would be expected for noise originating from microscopic fluctuators on the surface (*7*). At greater ion-electrode distances, the noise spectral density appears to approach a constant level, indicating that a different noise source is dominant there.

We further deploy the ability to detach the electrodes from the voltage sources when determining the axial heating rates and observe little effect. Comparing the yellow and purple lines in Fig. 3A, corresponding to measurements taken with and without trap detachment, we observe that most data points taken at the same value of *d* are within each other’s error bars. The deviation of the axial heating rates from the $d^{-4}$ scaling at large *d* may thus be caused by electromagnetic pickup in the wiring elements between the trap and the switches, or by direct electromagnetic interference (*7*).

Similarly, we measure and analyze the heating rates of the radial modes of motion, with the data presented alongside the axial heating rate data in Fig. 3A. For both magnetron and modified cyclotron modes, the heating rates are well above those observed using the axial mode. Assuming multiple participating noise sources as in the analysis of the axial heating rates, we again perform fits that reveal the strength of the various constituents (see Supplementary text and fig. S6). We find that the likely cause for the increased noise level observed using the radial modes is found in voltage fluctuations of technical origin in combination with differences in filtering between some of the electrodes. Those electrodes that carry the mode-coupling drive are equipped with filters of lower attenuation than all others and are thus likely to carry the most noise. These electrodes extend along the axial direction as can be seen in Fig. 1. As a consequence, they can cause no disturbing fields along *z* and further produce negligible heating at a symmetric point 152 μm above the surface, explaining the features of local minima visible in the fits in Fig. 3A. This technical noise is comparable in strength to the surface noise component, for which we obtain scaling exponents β=4.1±0.6 for the magnetron data and β=3.5±0.6 for the cyclotron data.

We further determine the dependence of the axial heating rates on the noise frequency, revealing the frequency-scaling exponent α. Such measurements are made by determining the axial heating rate ${\dot{\bar{n}}}_{z}$ at a range of frequencies $\omega_{z}$. We perform this at three different ion-electrode distances *d* with the results shown in Fig. 3B. At d=75 and 152 μm, the scaling exponent is consistent with α≈1.7 while α=0.6±0.3 is found at d≈450 μm. As noted in the discussion of the behavior of the axial heating rates with distance, the noise level at this most distant location deviates from the power-law predictions associated with surface noise. The fact that also the frequency-scaling behavior differs at this particular location is a further indication that different noise sources dominate depending on position. Last, it is worth pointing out that while many models predict 1/f scaling, corresponding to α=1, the bulk of experimental evidence (*7*) finds that α deviates from this expectation, with most measurements finding values in a wider range between 0 and 2.

The measured heating rates can be converted to noise spectral densities as described in Eqs. 4 and 5. Notably, we measure an axial heating rate of 1.6(4) phonons/min at a distance d≈450 μm, corresponding to a spectral density of 1.1(2) × 10^−16^ V^2^ m^2^ Hz^−1^. To our knowledge, this constitutes the lowest spectral density observed in a surface-electrode trap to date (*62*). This measurement also sets the limit for the sensitivity to electric fields in our apparatus to 10 nV/m/$\sqrt{\text{Hz}}$.

Further using the determined values of α to rescale the obtained spectral densities to a common measurement frequency ω=2π×1 MHz allows a comparison of our results to prior work in rf traps. Figure 3C shows the portion of our data that are dominated by surface noise, as well as results obtained in previous experimental efforts in rf traps with adjustable ion-electrode distance. The noise levels reported here are comparable to the lowest values found in other experiments and the distance-scaling exponent is well in line with previous experimental investigations in rf traps (*20*, *63*–*66*). It is apparent from this comparison that the absence of a high-voltage rf drive in our apparatus does not necessarily yield a reduction in noise beyond what is observed in rf traps.

We further use 3D transport to sample the heating rates of all motional modes on the same grid of positions used for the stray-field measurements, with the results shown in Fig. 3D. Besides the increase in heating rates when approaching the surface, some structure is also visible within the planes parallel to the electrodes. As similarly concluded using cantilever probes sampling at distances around 100 nm (*67*), surface noise can have considerable spatial variation, which may further depend on the frequency and polarization of interest. Focusing on the axial mode, the heating rates at d=75 μm increase by half when moving 100 μm along *z* while reducing to one-fourth when displaced by the same amount along *x*. Using the cyclotron and magnetron modes, increased noise is observed when displaced perpendicular to the axial direction; however, most of this can be accounted for by thermal and technical noise (see Supplementary text and fig. S6). This 3D spatial resolution paves the way for investigations into surface science where electric-field noise is probed depending on all three relevant variables ω, *d*, and *T* and involving multiple materials and surface constitutions.

### Sensing magnetic fields

In addition to electric field sensing, we use the sensitivity of the electron spin of the ion to probe magnetic fields. The splitting between the two qubit states depends on the magnetic field at approximately 28 GHz/T. We perform Rabi spectroscopy of the qubit transition with a microwave field close to 83.2 GHz and convert the frequency splitting to the magnetic field using the Breit-Rabi formula (*68*). Simultaneous with probing of the qubit frequency, we measure the Rabi frequency of the transition, thus also probing the microwave field strength perpendicular to the quantization axis.

**Fig. 4.**
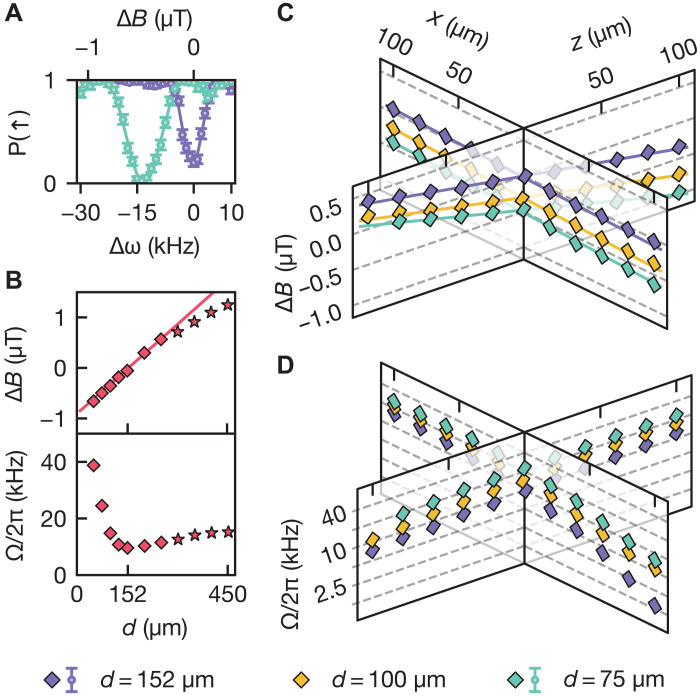
Sensing of magnetic fields. (**A**) Population P(↑) after applying a microwave pulse to an ion initially in ∣↑〉. Data in purple (turquoise) correspond to the ion being 152 μm (75 μm) from the surface. Frequency shifts are measured relative to the resonance frequency $\omega_{0}$ at position (0, 152 μm, 0). (**B**) Change in the magnetic field and Rabi rate of the microwave drive measured for several ion-electrode distances *d* between 50 and 450 μm. Star symbols indicate that *d* was not verified and no stray-field correction was applied. The solid line is a linear fit to the data with verified distance. (**C**) Magnetic field shifts within planes parallel to the trap surface at three heights. Solid lines are linear fits, revealing field gradients between −3.59 ± 0.06 and −5.26 ± 0.08 nT μm^−1^ along *z* and less than 0.74 ± 0.04 nT μm^−1^ along *x*. (**D**) Spatial dependence of the Rabi rate of the microwave drive. Error bars are smaller than the markers for (B) to (D).

Example data of spin-flip resonances, taken at positions (0, 152 μm, 0) and (0, 75 μm, 0), are shown in Fig. 4A, indicating a frequency shift as well as broadening due to a spatial variation in the microwave field strength. Given the spin coherence time of approximately 1.9 ms in our apparatus (*46*), the magnetic field is sensed at a sensitivity of around 2 nT/$\sqrt{\text{Hz}}$ (*69*). The sensitivity of trapped ions to magnetic fields can be greatly improved by increasing coherence times, for example, through improved shielding and active cancellation of external noise (*70*), as well as through dynamical decoupling methods (*39*), with the best result to date reaching a sensitivity of 4.6 pT/$\sqrt{\text{Hz}}$ (*39*).

Figure 4B shows the variation of both the static and microwave field strengths at (*x*, *z*) = (0, 0) as a function of the height of the ion above the surface. The magnetic field shows a gradient of 5.87 ± 0.16 nT μm^−1^, while the microwave field shows a rapid increase in strength toward the surface. In Fig. 4 (C and D), we show variations in the three planes at different heights above the surface. Here, we see that the static field shows a gradient mainly along *z*, while the microwave field strength is greatly reduced at position (−100 μm, 152 μm, 0). The measured static field gradients are about two orders of magnitude larger than the empty-bore magnet inhomogeneity specified by the manufacturer, with materials placed in the bore a likely cause of the field distortions. While the microwave radiation is expected to show standing-wave behavior along its propagation direction (*z*) due to a Faraday cage surrounding the trap, the rapid variation of the microwave field strength along the *x* and *y* directions are unexpected and likely caused by interaction of the microwave radiation with the metal surface. Such measurement capability may aid the design and characterization of near-field microwave devices (*53*, *71*, *72*).

## DISCUSSION

Herein, we have combined the sensitivity of trapped ions to electric and magnetic fields with the arbitrary positioning close to surfaces that are available in microfabricated Penning traps. Knowledge of the measured fields with 3D resolution provides a level of information that is not otherwise available to trapped-ion sensors. This has, for example, allowed us to extract the charge distribution on the trap surface as well as to unambiguously distinguish between various noise sources, such as noise stemming from a metallic surface and noise originating in technical equipment, a typical confounding factor in surface science measurements using trapped ions.

Integrating such a trapped-ion sensor with further in situ tools for surface probing and modification (*22*), together with enabling fast sample exchange, will result in a general-purpose apparatus for surface science (*67*) with quantum-limited sensitivity to electric fields. This platform can be enhanced by applying more advanced measurement techniques from the toolbox of quantum sensing, such as harnessing entanglement (*37*, *40*) and engineered quantum states (*73*, *74*).

We expect that our sensing platform will be a key enabler of the rapid improvements in material selection and validation of fabrication processes that are needed for reliable quantum devices. In particular, efforts at realizing scaled quantum computers consisting of any combination of physical systems are stifled by materials challenges (*1*). Testing the effects of surface treatments (*20*), terminations (*25*), or functionalizations (*24*, *75*) as well as advanced fabrication methods such as atomic-layer deposition (*76*, *77*) is possible using the proposed strategy.

## MATERIALS AND METHODS

### Achievable spatial resolution

In this work, we map electric and magnetic fields in intervals of 10 μm. However, the spatial resolution with which we position the ion in our apparatus is far larger and limited primarily by the DAC step size of 1.2 mV. The resulting level of control over displacement fields is about 1 V m^−1^ in the most sensitive direction (*y*), corresponding to positional shifts in steps of around 0.1 μm at typical settings. Detection of spatial displacements using a camera is the key mechanism enabling our method of measuring static electric fields, with a measurement accuracy of around 0.4 to 1.0 μm, depending on the imaging magnification available at a given ion position (see Supplementary text, in particular, fig. S2).

### Experimental sequence for the measurement of static electric fields

Our method of measurement relies on the fact that ions in Penning traps are confined at the null of the total electric potential experienced by the particle. If stray electric fields are present in the trap, a variation of the strength of the applied electric potential ϕ will displace the ion. The ion position is recorded on a camera as the applied curvature is changed. Homogeneous electric fields are further applied through the trap electrodes, allowing to spatially shift the ion. In an iterative process, these correction fields are varied until the ion position is independent of the curvature strength of the applied potential ϕ. Once this condition is reached, the stray fields are taken to be the opposite of the applied correction fields.

A measurement sequence starts by performing laser cooling and preparation of the bright state at the initial location to which the relevant laser beams are aligned. Subsequently, the ion is transported to a target location, where it is illuminated by a secondary detection laser beam and its fluorescence collected on a camera. Contrary to the usual fluorescence readout in our apparatus, no mode-coupling drive is applied during this detection pulse as the ion may be far removed from the field null of the drive. Otherwise, the ion may undergo substantial coherent motion despite the low amplitude of the drive of less than 20 mV.

Having chosen an electric potential with a certain curvature as well as candidate correction fields, the resulting position on the camera sensor is recorded as well as the shape of the point spread function. Distortions of the latter serve as a measure for displacements in the out-of-plane direction *y*, which is along the optical path of the detection system, thus causing defocusing effects. Last, the ion is returned to its original position and the measurement iterated until the effect of the stray fields is compensated. The uncertainty of the measurement is estimated by varying the correction fields around the determined stray field value. We observe that the relation between the correction field and the ion position as well as the focus size change at different rates depending on the curvature of the applied trapping potential and perform fits to the resulting data. Together with an estimate of the uncertainty of the ion position observed on the camera, the intersection of the fit lines yield estimates of the error of the stray field measurements. We perform such error estimations at a number of position throughout the sampled volume. Further detail is given in Supplementary text, in particular, in fig. S2).

### Experimental sequence for the measurement of electric-field noise

For the measurement of electric-field noise, the ion first undergoes Doppler cooling and resolved-sideband cooling (*46*), resulting in the motional mode of interest λ being cooled close to the ground state. This takes place at the initial location to which the laser beams for cooling and coherent manipulation are aligned. The ion is then also prepared in the bright qubit state. Transport to a target location follows, where the ion remains for the duration of a variable wait time during which electric-field noise resonantly heats the motional mode λ. After transport back to the original position, we probe a motional sideband of the qubit transition, followed by fluorescence detection of the spin state. The comparison of the excitation imbalance between the red and blue motional sidebands allows for the determination of the average phonon number ${\bar{n}}_{\lambda }$ in the mode (*6*).

To ensure accurate placement of the ion at the target location, we further compensate for the stray electric fields that were measured above the trap. In addition, we ensure that the motional frequency of the mode under investigation is equal at the starting and target locations. Last, transport is carried out in an adiabatic fashion, ensuring that no heating is caused by the transport sequence itself (*46*).

### Experimental sequence for the measurement of magnetic fields

The measurement sequence again consists of initial Doppler cooling and state preparation, followed by transport to a target location while canceling stray fields. There, a microwave pulse using the horn antenna (see Fig. 1) drives the qubit transition, followed by transport back to the original position and state detection. When measuring the shifts of the static magnetic field, we scan the frequency of the microwave radiation across the spin-flip resonance. In contrast, we fix the frequency to be on resonance when measuring the Rabi rate of the transition, while scanning the pulse duration. The experimental sequences are repeated 100 times.

## Supplementary Materials

### This PDF file includes:

Supplementary Text Figs. S1 to S8 References

## References

[1] N. P. de Leon, K. M. Itoh, D. Kim, K. K. Mehta, T. E. Northup, H. Paik, B. S. Palmer, N. Samarth, S. Sangtawesin, D. W. Steuerman, Materials challenges and opportunities for quantum computing hardware. Science 372, eabb2823 (2021). 33859004 10.1126/science.abb2823

[2] J. M. Obrecht, R. J. Wild, E. A. Cornell, Measuring electric fields from surface contaminants with neutral atoms. Phys. Rev. A 75, 062903 (2007).

[3] J. L. Garrett, J. Kim, J. N. Munday, Measuring the effect of electrostatic patch potentials in casimir force experiments. Phys. Rev. Res. 2, 023355 (2020).

[4] F. Antonucci, A. Cavalleri, R. Dolesi, M. Hueller, D. Nicolodi, H. B. Tu, S. Vitale, W. J. Weber, Interaction between stray electrostatic fields and a charged free-falling test mass. Phys. Rev. Lett. 108, 181101 (2012). 22681053 10.1103/PhysRevLett.108.181101

[5] W.-C. Dong, W.-H. Tan, Z.-J. An, H. Huang, L. Zhu, Y.-J. Tan, T.-Y. Long, C.-G. Shao, S.-Q. Yang, Coupling effect of vibrations and residual electrostatic force in short-range gravitational experiments. Phys. Rev. Appl. 20, 054046 (2023).

[6] C. Monroe, D. M. Meekhof, B. E. King, S. R. Jefferts, W. M. Itano, D. J. Wineland, P. Gould, Resolved-sideband Raman cooling of a bound atom to the 3D zero-point energy. Phys. Rev. Lett. 75, 4011–4014 (1995). 10059792 10.1103/PhysRevLett.75.4011

[7] M. Brownnutt, M. Kumph, P. Rabl, R. Blatt, Ion-trap measurements of electric-field noise near surfaces. Rev. Mod. Phys. 87, 1419–1482 (2015).

[8] J. D. Carter, J. D. D. Martin, Coherent manipulation of cold Rydberg atoms near the surface of an atom chip. Phys. Rev. A 88, 043429 (2013).

[9] D. Davtyan, S. Machluf, M. L. Soudijn, J. B. Naber, N. J. van Druten, H. B. van Linden van den Heuvell, R. J. C. Spreeuw, Controlling stray electric fields on an atom chip for experiments on Rydberg atoms. Phys. Rev. A 97, 023418 (2018).

[10] S. E. de Graaf, L. Faoro, L. B. Ioffe, S. Mahashabde, J. J. Burnett, T. Lindström, S. E. Kubatkin, A. V. Danilov, A. Y. Tzalenchuk, Two-level systems in superconducting quantum devices due to trapped quasiparticles. Sci. Adv. 6, eabc5055 (2020). 33355127 10.1126/sciadv.abc5055 PMC11206451

[11] M. Kim, H. J. Mamin, M. H. Sherwood, K. Ohno, D. D. Awschalom, D. Rugar, Decoherence of near-surface nitrogen-vacancy centers due to electric field noise. Phys. Rev. Lett. 115, 087602 (2015). 26340208 10.1103/PhysRevLett.115.087602

[12] P. L. Ocola, I. Dimitrova, B. Grinkemeyer, E. Guardado-Sanchez, T. Dordević, P. Samutpraphoot, V. Vuletić, M. D. Lukin, Control and entanglement of individual Rydberg atoms near a nanoscale device. Phys. Rev. Lett. 132, 113601 (2024). 38563952 10.1103/PhysRevLett.132.113601

[13] T. Thiele, S. Filipp, J. A. Agner, H. Schmutz, J. Deiglmayr, M. Stammeier, P. Allmendinger, F. Merkt, A. Wallraff, Manipulating Rydberg atoms close to surfaces at cryogenic temperatures. Phys. Rev. A 90, 013414 (2014).

[14] S. Sangtawesin, B. L. Dwyer, S. Srinivasan, J. J. Allred, L. V. H. Rodgers, K. De Greve, A. Stacey, N. Dontschuk, K. M. O’Donnell, D. Hu, D. A. Evans, C. Jaye, D. A. Fischer, M. L. Markham, D. J. Twitchen, H. Park, M. D. Lukin, N. P. de Leon, Origins of diamond surface noise probed by correlating single-spin measurements with surface spectroscopy. Phys. Rev. X 9, 031052 (2019).

[15] K.-Y. Lin, G. H. Low, I. L. Chuang, Effects of electrode surface roughness on motional heating of trapped ions. Phys. Rev. A 94, 013418 (2016).

[16] C. Müller, J. H. Cole, J. Lisenfeld, Towards understanding two-level-systems in amorphous solids: Insights from quantum circuits. Rep. Prog. Phys. 82, 124501 (2019). 31404914 10.1088/1361-6633/ab3a7e

[17] E. Kim, A. Safavi-Naini, D. A. Hite, K. S. McKay, D. P. Pappas, P. F. Weck, H. R. Sadeghpour, Electric-field noise from carbon-adatom diffusion on a au(110) surface: First-principles calculations and experiments. Phys. Rev. A 95, 033407 (2017).

[18] J. Lisenfeld, A. Bilmes, A. Megrant, R. Barends, J. Kelly, P. Klimov, G. Weiss, J. M. Martinis, A. V. Ustinov, Electric field spectroscopy of material defects in transmon qubits. npj Quantum Inf. 5, 105 (2019).

[19] J. M. McGuirk, D. M. Harber, J. M. Obrecht, E. A. Cornell, Alkali-metal adsorbate polarization on conducting and insulating surfaces probed with bose-einstein condensates. Phys. Rev. A 69, 062905 (2004).

[20] J. A. Sedlacek, J. Stuart, D. H. Slichter, C. D. Bruzewicz, R. McConnell, J. M. Sage, J. Chiaverini, Evidence for multiple mechanisms underlying surface electric-field noise in ion traps. Phys. Rev. A 98, 063430 (2018).

[21] D. A. Hite, Y. Colombe, A. C. Wilson, K. R. Brown, U. Warring, R. Jördens, J. D. Jost, K. S. McKay, D. P. Pappas, D. Leibfried, D. J. Wineland, 100-fold reduction of electric-field noise in an ion trap cleaned with in situ argon-ion-beam bombardment. Phys. Rev. Lett. 109, 103001 (2012). 23005284 10.1103/PhysRevLett.109.103001

[22] D. A. Hite, K. S. McKay, D. P. Pappas, Surface science motivated by heating of trapped ions from the quantum ground state. New J. Phys. 23, 103028 (2021). 10.1088/1367-2630/ac2c2c PMC10938442 38487593

[23] M. Mergenthaler, C. Müller, M. Ganzhorn, S. Paredes, P. Müller, G. Salis, V. P. Adiga, M. Brink, M. Sandberg, J. B. Hertzberg, S. Filipp, A. Fuhrer, Effects of surface treatments on flux tunable transmon qubits. npj Quantum Inf. 7, 157 (2021).

[24] M. Alghadeer, A. Banerjee, K. Lee, H. Hussein, H. Fariborzi, S. Rao, Mitigating coherent loss in superconducting circuits using molecular self-assembled monolayers. Sci. Rep. 14, 27340 (2024). 39521838 10.1038/s41598-024-77227-7 PMC11550477

[25] P. Chrostoski, H. R. Sadeghpour, D. H. Santamore, Electric noise spectra of a near-surface nitrogen-vacancy center in diamond with a protective layer. Phys. Rev. Appl. 10, 064056 (2018).

[26] S. Lin, C. Weng, J. Wang, Y. Guo, Y. Yang, J. Zhao, P. Ma, Y. Chen, L. Lou, W. Zhu, G. Wang, Diamond surface electric-field noise detection using shallow nitrogen-vacancy centers. Phys. Rev. B 106, 165406 (2022).

[27] Y. Tao, P. Navaretti, R. Hauert, U. Grob, M. Poggio, C. L. Degen, Permanent reduction of dissipation in nanomechanical si resonators by chemical surface protection. Nanotechnology 26, 465501 (2015). 26501931 10.1088/0957-4484/26/46/465501

[28] J. A. Sedlacek, J. Stuart, W. Loh, R. McConnell, C. D. Bruzewicz, J. M. Sage, J. Chiaverini, Method for determination of technical noise contributions to ion motional heating. J. Appl. Phys. 124, 214904 (2018).

[29] A. Berzins, M. Saleh Ziabari, Y. Silani, I. Fescenko, J. T. Damron, J. F. Barry, A. Jarmola, P. Kehayias, B. A. Richards, J. Smits, V. M. Acosta, Impact of microwave phase noise on diamond quantum sensing. Phys. Rev. Res. 6, 043148 (2024). 10.1103/physrevresearch.6.043148 PMC12524190 41103975

[30] D. Budker, M. Romalis, Optical magnetometry. Nat. Phys. 3, 227–234 (2007).

[31] M. Block, B. Kobrin, A. Jarmola, S. Hsieh, C. Zu, N. Figueroa, V. Acosta, J. Minguzzi, J. Maze, D. Budker, N. Yao, Optically enhanced electric field sensing using nitrogen-vacancy ensembles. Phys. Rev. Appl. 16, 024024 (2021).

[32] T. Wolf, P. Neumann, K. Nakamura, H. Sumiya, T. Ohshima, J. Isoya, J. Wrachtrup, Subpicotesla diamond magnetometry. Phys. Rev. X 5, 041001 (2015).

[33] M. Simmonds, W. Fertig, R. Giffard, Performance of a resonant input squid amplifier system. IEEE Trans. Magn. 15, 478–481 (1979).

[34] T. Bagci, A. Simonsen, S. Schmid, L. G. Villanueva, E. Zeuthen, J. Appel, J. M. Taylor, A. Sørensen, K. Usami, A. Schliesser, E. S. Polzik, Optical detection of radio waves through a nanomechanical transducer. Nature 507, 81–85 (2014). 24598636 10.1038/nature13029

[35] E. Marchiori, L. Ceccarelli, N. Rossi, L. Lorenzelli, C. L. Degen, M. Poggio, Nanoscale magnetic field imaging for 2D materials. Nat. Rev. Phys. 4, 49–60 (2022).

[36] Z. Qiu, A. Hamo, U. Vool, T. X. Zhou, A. Yacoby, Nanoscale electric field imaging with an ambient scanning quantum sensor microscope. npj Quantum Inf. 8, 107 (2022).

[37] T. Ruster, H. Kaufmann, M. A. Luda, V. Kaushal, C. T. Schmiegelow, F. Schmidt-Kaler, U. G. Poschinger, Entanglement-based dc magnetometry with separated ions. Phys. Rev. X 7, 031050 (2017).

[38] A. R. Vasquez, C. Mordini, C. Vernière, M. Stadler, M. Malinowski, C. Zhang, D. Kienzler, K. K. Mehta, J. P. Home, Control of an atomic quadrupole transition in a phase-stable standing wave. Phys. Rev. Lett. 130, 133201 (2023). 37067320 10.1103/PhysRevLett.130.133201

[39] I. Baumgart, J.-M. Cai, A. Retzker, M. B. Plenio, C. Wunderlich, Ultrasensitive magnetometer using a single atom. Phys. Rev. Lett. 116, 240801 (2016). 27367376 10.1103/PhysRevLett.116.240801

[40] D. Leibfried, M. D. Barrett, T. Schaetz, J. Britton, J. Chiaverini, W. M. Itano, J. D. Jost, C. Langer, D. J. Wineland, Toward heisenberg-limited spectroscopy with multiparticle entangled states. Science 304, 1476–1478 (2004). 15178794 10.1126/science.1097576

[41] K. A. Gilmore, M. Affolter, R. J. Lewis-Swan, D. Barberena, E. Jordan, A. M. Rey, J. J. Bollinger, Quantum-enhanced sensing of displacements and electric fields with two-dimensional trapped-ion crystals. Science 373, 673–678 (2021). 34353950 10.1126/science.abi5226

[42] H. Wu, G. D. Mitts, C. Z. C. Ho, J. A. Rabinowitz, E. R. Hudson, Wideband electric field quantum sensing via motional raman transitions. Nat. Phys. 21, 380–385 (2025).

[43] P. Kaufmann, T. F. Gloger, D. Kaufmann, M. Johanning, C. Wunderlich, High-fidelity preservation of quantum information during trapped-ion transport. Phys. Rev. Lett. 120, 010501 (2018). 29350951 10.1103/PhysRevLett.120.010501

[44] K. R. Brown, J. Chiaverini, J. M. Sage, H. Häffner, Materials challenges for trapped-ion quantum computers. Nat. Rev. Mater. 6, 892–905 (2021).

[45] S. Jain, J. Alonso, M. Grau, J. P. Home, Scalable arrays of micro-penning traps for quantum computing and simulation. Phys. Rev. X 10, 031027 (2020).

[46] S. Jain, T. Sägesser, P. Hrmo, C. Torkzaban, M. Stadler, R. Oswald, C. Axline, A. Bautista-Salvador, C. Ospelkaus, D. Kienzler, J. Home, Penning micro-trap for quantum computing. Nature 627, 510–514 (2024). 38480890 10.1038/s41586-024-07111-x PMC10954548

[47] M. Harlander, M. Brownnutt, W. Hänsel, R. Blatt, Trapped-ion probing of light-induced charging effects on dielectrics. New J. Phys. 12, 093035 (2010).

[48] S. Auchter, C. Axline, C. Decaroli, M. Valentini, L. Purwin, R. Oswald, R. Matt, E. Aschauer, Y. Colombe, P. Holz, T. Monz, R. Blatt, P. Schindler, C. Rössler, J. Home, Industrially microfabricated ion trap with 1 eV trap depth. Quantum Sci. Technol. 7, 035015 (2022).

[49] T. C. Leung, C. L. Kao, W. S. Su, Y. J. Feng, C. T. Chan, Relationship between surface dipole, work function and charge transfer: Some exceptions to an established rule. Phys. Rev. B 68, 195408 (2003).

[50] K. Bian, W. Zheng, X. Zeng, X. Chen, R. Stöhr, A. Denisenko, S. Yang, J. Wrachtrup, Y. Jiang, Nanoscale electric-field imaging based on a quantum sensor and its charge-state control under ambient condition. Nat. Commun. 12, 2457 (2021). 33911073 10.1038/s41467-021-22709-9 PMC8080810

[51] C. Wagner, M. F. B. Green, M. Maiworm, P. Leinen, T. Esat, N. Ferri, N. Friedrich, R. Findeisen, A. Tkatchenko, R. Temirov, F. S. Tautz, Quantitative imaging of electric surface potentials with single-atom sensitivity. Nat. Mater. 18, 853–859 (2019). 31182779 10.1038/s41563-019-0382-8 PMC6656579

[52] S. Zhang, K. Bian, Y. Jiang, Perspective: Nanoscale electric sensing and imaging based on quantum sensors. Quantum Front. 2, 19 (2023).

[53] U. Warring, C. Ospelkaus, Y. Colombe, K. R. Brown, J. M. Amini, M. Carsjens, D. Leibfried, D. J. Wineland, Techniques for microwave near-field quantum control of trapped ions. Phys. Rev. A 87, 013437 (2013).

[54] F. R. Ong, K. Schüppert, P. Jobez, M. Teller, B. Ames, D. A. Fioretto, K. Friebe, M. Lee, Y. Colombe, R. Blatt, T. E. Northup, Probing surface charge densities on optical fibers with a trapped ion. New J. Phys. 22, 063018 (2020).

[55] D. Wineland, C. Monroe, W. Itano, D. Leibfried, B. King, D. Meekhof, Experimental issues in coherent quantum-state manipulation of trapped atomic ions. J. Res. Natl. Inst. Stand. Technol. 103, 259–328 (1998). 28009379 10.6028/jres.103.019 PMC4898965

[56] G. H. Low, P. F. Herskind, I. L. Chuang, Finite-geometry models of electric field noise from patch potentials in ion traps. Phys. Rev. A 84, 053425 (2011).

[57] M. Kumph, P. Holz, K. Langer, M. Meraner, M. Niedermayr, M. Brownnutt, R. Blatt, Operation of a planar-electrode ion-trap array with adjustable rf electrodes. New J. Phys. 18, 023047 (2016).

[58] L. Martinetz, K. Hornberger, B. A. Stickler, Surface-induced decoherence and heating of charged particles. PRX Quantum 3, 030327 (2022).

[59] A. Safavi-Naini, P. Rabl, P. F. Weck, H. R. Sadeghpour, Microscopic model of electric-field-noise heating in ion traps. Phys. Rev. A 84, 023412 (2011).

[60] B. L. Foulon, K. G. Ray, C.-E. Kim, Y. Liu, B. M. Rubenstein, V. Lordi, 1/ωelectric-field noise in surface ion traps from correlated adsorbate dynamics. Phys. Rev. A 105, 013107 (2022).

[61] C. Noel, M. Berlin-Udi, C. Matthiesen, J. Yu, Y. Zhou, V. Lordi, H. Häffner, Electric-field noise from thermally activated fluctuators in a surface ion trap. Phys. Rev. A 99, 063427 (2019).

[62] S. A. King, L. J. Spieß, P. Micke, A. Wilzewski, T. Leopold, J. R. Crespo López-Urrutia, P. O. Schmidt, Algorithmic ground-state cooling of weakly coupled oscillators using quantum logic. Phys. Rev. X 11, 041049 (2021).

[63] I. A. Boldin, A. Kraft, C. Wunderlich, Measuring anomalous heating in a planar ion trap with variable ion-surface separation. Phys. Rev. Lett. 120, 023201 (2018). 29376708 10.1103/PhysRevLett.120.023201

[64] J. A. Sedlacek, A. Greene, J. Stuart, R. McConnell, C. D. Bruzewicz, J. M. Sage, J. Chiaverini, Distance scaling of electric-field noise in a surface-electrode ion trap. Phys. Rev. A 97, 020302 (2018).

[65] D. An, C. Matthiesen, E. Urban, H. Häffner, Distance scaling and polarization of electric-field noise in a surface ion trap. Phys. Rev. A 100, 063405 (2019).

[66] K. S. McKay, D. A. Hite, P. D. Kent, S. Kotler, D. Leibfried, D. H. Slichter, A. C. Wilson, D. P. Pappas, Measurement of electric-field noise from interchangeable samples with a trapped-ion sensor. Phys. Rev. A 104, 052610 (2021). 10.1103/PhysRevA.104.052610 PMC11194989 38915757

[67] M. Héritier, R. Pachlatko, Y. Tao, J. M. Abendroth, C. L. Degen, A. Eichler, Spatial correlation between fluctuating and static fields over metal and dielectric substrates. Phys. Rev. Lett. 127, 216101 (2021). 34860104 10.1103/PhysRevLett.127.216101

[68] N. Shiga, W. M. Itano, J. J. Bollinger, Diamagnetic correction to the ^9^Be^+^ ground-state hyperfine constant. Phys. Rev. A 84, 012510 (2011).

[69] C. L. Degen, F. Reinhard, P. Cappellaro, Quantum sensing. Rev. Mod. Phys. 89, 035002 (2017).

[70] T. Ruster, C. T. Schmiegelow, H. Kaufmann, C. Warschburger, F. Schmidt-Kaler, U. G. Poschinger, A long-lived zeeman trapped-ion qubit. Appl. Phys. B 122, 254 (2016).

[71] H. Q. Fan, S. Kumar, R. Daschner, H. Kübler, J. P. Shaffer, Subwavelength microwave electric-field imaging using Rydberg atoms inside atomic vapor cells. Opt. Lett. 39, 3030–3033 (2014). 24978265 10.1364/OL.39.003030

[72] M. A. Weber, C. Löschnauer, J. Wolf, M. F. Gely, R. K. Hanley, J. F. Goodwin, C. J. Ballance, T. P. Harty, D. M. Lucas, Cryogenic ion trap system for high-fidelity near-field microwave-driven quantum logic. Quantum Sci. Technol. 9, 015007 (2024).

[73] S. C. Burd, R. Srinivas, J. J. Bollinger, A. C. Wilson, D. J. Wineland, D. Leibfried, D. H. Slichter, D. T. C. Allcock, Quantum amplification of mechanical oscillator motion. Science 364, 1163–1165 (2019). 31221854 10.1126/science.aaw2884 PMC11566721

[74] A. R. Milne, C. Hempel, L. Li, C. L. Edmunds, H. J. Slatyer, H. Ball, M. R. Hush, M. J. Biercuk, Quantum oscillator noise spectroscopy via displaced cat states. Phys. Rev. Lett. 126, 250506 (2021). 34241523 10.1103/PhysRevLett.126.250506

[75] J. C. Thomas, J. J. Schwartz, J. N. Hohman, S. A. Claridge, H. S. Auluck, A. C. Serino, A. M. Spokoyny, G. Tran, K. F. Kelly, C. A. Mirkin, J. Gilles, S. J. Osher, P. S. Weiss, Defect-tolerant aligned dipoles within two-dimensional plastic lattices. ACS Nano 9, 4734–4742 (2015). 25867638 10.1021/acsnano.5b01329

[76] R. Lu, A. J. Elliot, L. Wille, B. Mao, S. Han, J. Z. Wu, J. Talvacchio, H. M. Schulze, R. M. Lewis, D. J. Ewing, H. F. Yu, G. M. Xue, S. P. Zhao, Fabrication of Nb/Al_2_O_3_/Nb josephson junctions using in situ magnetron sputtering and atomic layer deposition. IEEE Trans. Appl. Supercond. 23, 1100705–1100705 (2013).

[77] R. Kumar, S. Mahajan, F. Donaldson, S. Dhomkar, H. J. Lancaster, C. Kalha, A. A. Riaz, Y. Zhu, C. A. Howard, A. Regoutz, J. J. L. Morton, Stability of Near-Surface nitrogen vacancy centers using dielectric surface passivation. ACS Photonics 11, 1244–1251 (2024). 38523744 10.1021/acsphotonics.3c01773 PMC10958592

[78] C. Mordini, F. Lancellotti, V. Negnevitsky, M. Marinelli, R. Oswald, T. Saegesser, pytrans (v2.1.0+a). Zenodo (2023); 10.5281/zenodo.10204606.

[79] G. Bradski, The OpenCV Library. Dr. Dobb’s J. Softw. Tools 25, 120–126 (2000).

[80] D. B. Murphy, M. W. Davidson, “Diffraction and spatial resolution” in *Fundamentals of Light Microscopy and Electronic Imaging* (John Wiley & Sons, Ltd, ed. 2, 2012), chap. 6, p. 109.

[81] S. X. Wang, G. Hao Low, N. S. Lachenmyer, Y. Ge, P. F. Herskind, I. L. Chuang, Laser-induced charging of microfabricated ion traps. J. Appl. Phys. 110, 104901 (2011).

[82] W. Lee, D. Chung, H. Jeon, B. Cho, K. Choi, S. Yoo, C. Jung, J. Jeong, C. Kim, D.-I. D. Cho, T. Kim, Photoinduced charge-carrier dynamics in a semiconductor-based ion trap investigated via motion-sensitive qubit transitions. Phys. Rev. A 109, 043106 (2024).

[83] A. Härter, A. Krükow, A. Brunner, J. Hecker Denschlag, Long-term drifts of stray electric fields in a paul trap. Appl. Phys. B 114, 275–281 (2014).

[84] S. C. Doret, J. M. Amini, K. Wright, C. Volin, T. Killian, A. Ozakin, D. Denison, H. Hayden, C.-S. Pai, R. E. Slusher, A. W. Harter, Controlling trapping potentials and stray electric fields in a microfabricated ion trap through design and compensation. New J. Phys. 14, 073012 (2012).

[85] S. Narayanan, N. Daniilidis, S. A. Möller, R. Clark, F. Ziesel, K. Singer, F. Schmidt-Kaler, H. Häffner, Electric field compensation and sensing with a single ion in a planar trap. J. Appl. Phys. 110, 114909 (2011).

[86] A. Tauschinsky, R. M. T. Thijssen, S. Whitlock, H. B. van Linden van den Heuvell, R. J. C. Spreeuw, Spatially resolved excitation of Rydberg atoms and surface effects on an atom chip. Phys. Rev. A 81, 063411 (2010).

[87] J. A. Sedlacek, E. Kim, S. T. Rittenhouse, P. F. Weck, H. R. Sadeghpour, J. P. Shaffer, Electric field cancellation on quartz by Rb adsorbate-induced negative electron affinity. Phys. Rev. Lett. 116, 133201 (2016). 27081976 10.1103/PhysRevLett.116.133201

[88] T. A. Savard, K. M. O’Hara, J. E. Thomas, Laser-noise-induced heating in far-off resonance optical traps. Phys. Rev. A 56, R1095–R1098 (1997).

[89] D. Leibrandt, B. Yurke, R. Slusher, Modeling ion trap thermal noise decoherence. Quant. Inf. Comput. 7, 052–072 (2007).

