# Supplementary material for: A three-dimensional scanning trapped-ion probe
Source: Sci Adv. 2026 Jun 19;12(25):eaec0794. doi: 10.1126/sciadv.aec0794 (PMC13281824; doi:10.1126/sciadv.aec0794)
Supplement: Supplementary file 1 — Supplementary Text Figs. S1 to S8 References [file sciadv.aec0794_sm.pdf]

Science Advances
AAAS

Supplementary Materials for

A three-dimensional scanning trapped-ion probe

Tobias Sägesser *et al.*

Corresponding author: Tobias Sägesser, tobiass@phys.ethz.ch

Sci. Adv. **12**, eaec0794 (2026)
DOI: 10.1126/sciadv.aec0794

This PDF file includes:

Supplementary Text
Figs. S1 to S8
References

Supplementary Text

Transport and experimental sequences

The electrical potentials needed in conjunction with the magnetic field for trapping an ion at a particular location $\mathbf{r}_0 = (x_0, y_0, z_0)$ are generated by applying suitable voltages to the 25 independent electrodes of the trap chip. Using the software package PYTRANS (*78*), which applies convex optimization techniques, we calculate voltages that approximate a cylindrically symmetric electrical potential

$$\phi = \frac{m\omega_z^2}{2e}\left((z - z_0)^2 - \frac{(x - x_0)^2 + (y - y_0)^2}{2}\right) , \tag{S1}$$

where m is the ion mass and ω_z is the axial motional frequency along the direction z along the magnetic field. When performing ion transport, we calculate sequences of potentials such that the equilibrium position is varied in steps of 1 µm. Interpolation of the resulting voltage sequence leads to a smooth translation of the trap and to low-excitation transport of the ion. We furthermore calculate sets of voltages which produce purely electric fields at any desired location, with no potential curvature. Such fields are used for counteracting stray electric fields.

The speed of transport in our apparatus is severely restricted due to strong low-pass filtering applied to the trap electrodes, with the strongest filtering applied to the five strip electrodes closest to the ion with a cutoff frequency of approximately 7 kHz. The execution time of transport lies between 1 ms and 8 ms depending on the distance of displacement, corresponding to speeds between 2–6 $\mathrm{cm\,s^{-1}}$. At these speeds, we do not observe excitation of the motional modes during transport beyond what is expected from the heating rates at the respective ion–electrode distances (*46*).

Please refer to Fig. 1 for a depiction of the trap chip and the arrangement of the trap wiring, voltage sources, laser beams and the microwave source used to perform the experiments.

In this work, an experimental sequence always starts by Doppler cooling of the ion at the "cooling" location (0, 152 µm, 0) using the "Detection I" laser beam. Simultaneous to the cooling pulse, we apply an oscillating voltage at the bare cyclotron frequency $\omega_c = 2\pi \times 5.118$ MHz to the axialization electrodes (colored in beige in Fig. 1). The resulting coupling between the two radial modes of motion assists in cooling of the magnetron mode (*46*). Subsequently, the ion is always prepared in the bright state using the "Repump" beam. A transport sequence is then applied which brings the ion to a target location. Any path can be taken in principle and we choose to always

first move along the out-of-plane direction, bringing the ion to its target ion–electrode distance. Subsequently, we displace it in the x or z direction.

Imaging apparatus and calibration of the ion–electrode distance

We collect the ion fluorescence with a 0.55 NA Schwarzschild objective. Two lenses focus the imaging light onto an electron-multiplying charge-coupled device (EMCCD) camera or photomultiplier tube (PMT), where the second lens can be moved along the optical axis in order to bring ions into focus provided that the electrode–ion distance is smaller than approximately 300 µm. A to-scale view of the imaging components is provided in fig. S1a. Choosing from a variety of focal lengths of the movable lens L2 allows selecting the range of working distances across which an ion can be re-focused, as well as choosing the magnification. Maximizing the magnification is beneficial for measurements of static electric fields based on the detection of positional shifts. Ray-tracing simulations showing the relationship between the placement of the second lens and the resulting working distance and magnification are found in fig. S1b for the three sets of lenses used in our apparatus.

We use the relationship between lens placement and the resulting position of the focus to verify the ion-electrode distance. First, an absolute position reference is obtained by moving the ion to a known ion–electrode distance, in this case to the field null of the axialization electrodes, which we simulate to be at a height of 152 µm above the surface. If the ion is not at the field null, applying an oscillatory voltage at the cyclotron frequency leads to resonant excitation of the ion motion. A subsequent detection pulse registers a loss in fluorescence. We displace the ion until this excitation becomes minimal. Bringing the ion into focus and then measuring the position of L2 yields the working distance corresponding to an ion-electrode distance of $d = 152$ µm, marked by a star symbol in fig. S1b.

To calibrate other ion-electrode distances, we transport an ion to a desired nominal trap height as calculated using the electrostatic model of the trap, while correcting for stray fields. A pulse of the detection laser illuminates the ion and the resulting fluorescence is imaged. The movable lens L2 is placed such that the ion appears focused, with the resulting lens position translating to an estimate of the working distance as per the graphs in fig. S1b. The shift compared to the reference at $d = 152$ µm yields an estimate for the position of the ion relative to the surface. As plotted in

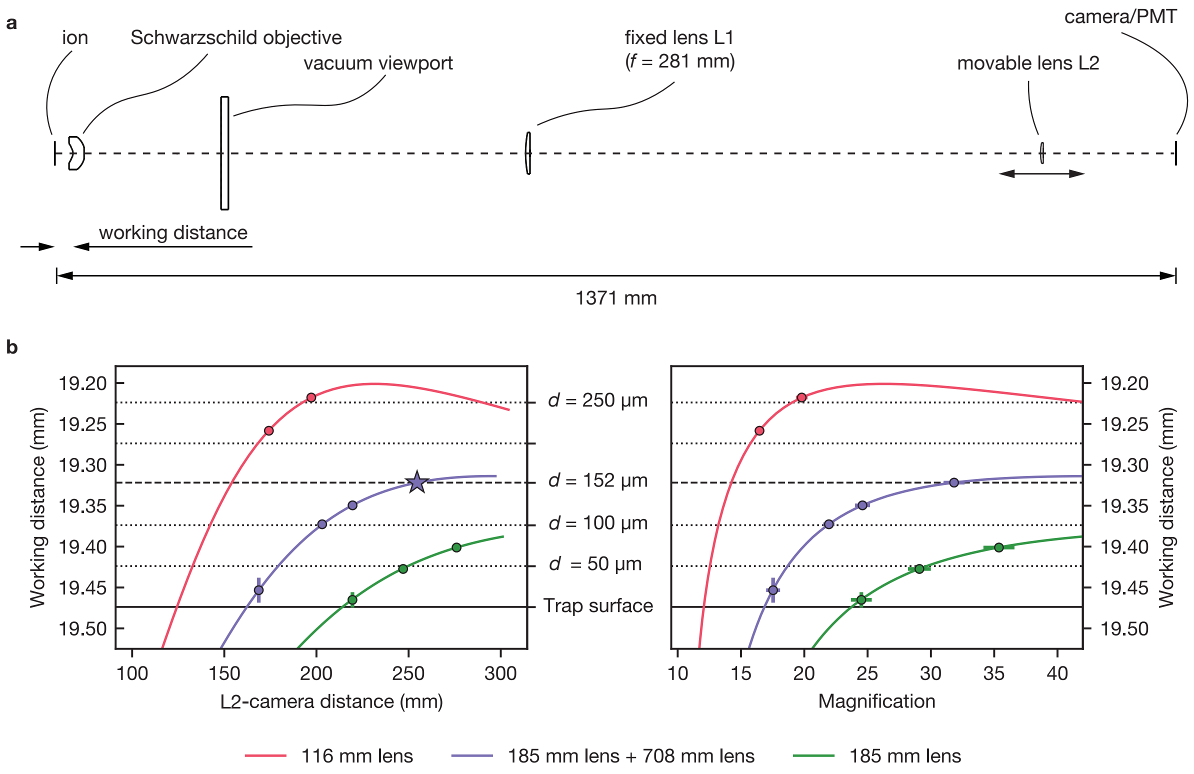

Figure S1: **Imaging system and ion–electrode distance calibration. a.** Schematic view of the imaging system. From left to right, fluorescence light from the ion is collected by a Schwarzschild objective, passes a viewport, and is then focused onto an EMCCD camera or a PMT by a fixed and a movable lens. **b.** Relationship between the position of the movable lens L2, the working distance at which the imaging focus occurs, and the resulting magnification. The dashed line as well as the star marker indicate the reference working distance found by moving the ion onto the rf null at d = 152 µm. The dotted lines show various ion-electrode distances relative to this reference, with the solid black line marking the expected location of the trap surface. Solid lines are ray-tracing simulations, where the three different focal lengths of L2 used in the experiment are chosen.

fig. S1b, we carry out this procedure for nominal trap heights of 50, 75, 100, 125, 152, 210 and 256 μm. At each position, we choose a suitable focal length of the movable lens L2 such that the magnification is maximized. Measured ion-electrode distances are 48 ± 4, 73 ± 4, 101 ± 4, 124 ± 3, 152 ± 0, 215 ± 4 and 255 ± 4 μm, where error bars take into account uncertainties in the placement of all the imaging components, as well as an estimate of the error in finding the true focus. Two further data points are taken by illuminating the trap surface and bringing it into focus. Using this implementation of the imaging system, the ion cannot be imaged if placed at ion–electrode distances greater than ≈ 300 μm. Data presented in this work with the ion at greater heights is thus given without visual confirmation of the location, as well as without stray-field correction.

Measurement of static electric fields

Due to the charged nature of trapped ions, external electric fields cause shifts in the position of an ion, which can be used to measure the field strength. We assume an ion to be trapped in the electrical potential $\phi_{\mathrm{app}}(\mathbf{r})$ deliberately applied through the electrodes, superposed with an additional stray potential $\phi_{\mathrm{stray}}(\mathbf{r})$, for example due to charging of the trap surface. Through electrostatic simulations, $\phi_{\mathrm{app}}(\mathbf{r})$ is calculated to confine an ion at a desired position $\mathbf{r}^*$ in the absence of stray electric fields, fulfilling $\nabla\phi_{\mathrm{app}}(\mathbf{r}^*) = 0$. Adding the field due to ϕ_{stray} will displace the ion from $\mathbf{r}^*$, with the shift determined by the magnitude of the curvatures of ϕ_{app}.

By minimizing the position shifts when scaling the curvature of the applied potential, the stray field can be determined. To this end, we calculate sets of voltages that produce purely electric fields at a given position above the trap chip and use these to spatially translate the ion. We set an initial correction field $\mathbf{E_1} = 0$ and translate the ion to a target location where the applied trapping potential is $f_{\mathrm{ax},1}^2 \cdot \phi_{\mathrm{app}}$, with $f_{\mathrm{ax},1}^2$ a scaling factor. Then, fluorescence detection is performed and the resulting position $\mathbf{r_0}$ of the ion on the camera sensor recorded. Subsequently, we scale the applied potential by a different factor $f_{\mathrm{ax},2}^2$ and additionally apply a correction field $\mathbf{E_2}$, chosen such that the ion is again found at $\mathbf{r_0}$. For both situations, the condition on the equilibrium position $\mathbf{r_0}$ is given by

$$\begin{aligned} \nabla\phi_{\mathrm{stray}}(\mathbf{r_0}) + (f_{\mathrm{ax},1})^2 \cdot \nabla\phi_{\mathrm{app}}(\mathbf{r_0}) - \mathbf{E_1} = 0 \\ \nabla\phi_{\mathrm{stray}}(\mathbf{r_0}) + (f_{\mathrm{ax},2})^2 \cdot \nabla\phi_{\mathrm{app}}(\mathbf{r_0}) - \mathbf{E_2} = 0\,. \end{aligned} \tag{S2}$$

We take ϕ_{app} to be a trapping potential resulting in an axial motional frequency of $\omega_z/2\pi =$

1 MHz. Thus, $f_{\mathrm{ax,i}}$ can be interpreted as the nominal axial frequency in multiples of a megahertz.

In this way, we can find both the stray field at position $\mathbf{r_0}$ and the gradient of the applied potential by rewriting eq. S2 to find

$$\begin{aligned}\nabla\phi_{\mathrm{app}}(\mathbf{r_0}) &= \frac{\mathbf{E_2} - \mathbf{E_1}}{f_{\mathrm{ax,2}}^2 - f_{\mathrm{ax,1}}^2} \\ \nabla\phi_{\mathrm{stray}}(\mathbf{r_0}) &= -\frac{f_{\mathrm{ax,1}}^2\mathbf{E_2} - f_{\mathrm{ax,2}}^2\mathbf{E_1}}{f_{\mathrm{ax,2}}^2 - f_{\mathrm{ax,1}}^2}\ .\end{aligned} \tag{S3}$$

In a typical situation, one would find that the gradient of the applied potential $\nabla\phi_{\mathrm{app}}(\mathbf{r_0})$ is not zero, meaning that the position $\mathbf{r_0}$ does not coincide with the true trap center $\mathbf{r}^*$. In this case, the measurement is restarted while incorporating the correction field by setting $\mathbf{E_1} = -\nabla\phi_{\mathrm{stray}}(\mathbf{r_0})$. By iterating this process, we eventually reach the position where $\mathbf{E_2} = \mathbf{E_1}$ and thus $\nabla\phi_{\mathrm{app}}(\mathbf{r_0}) = 0$.

In this way, we both calibrate the positioning of the ion by removing any shifts due to stray fields as well as measuring said fields. Translating the ion over a grid of positions allows to create a map of the stray field from which the stray potential can be reconstructed.

While the position of the ion in the directions perpendicular to the optical axis of the imaging apparatus, x and z, can be easily read by determining the center coordinates of the point-spread function observed on the camera sensor, obtaining information about the position along the imaging axis is more challenging. Images of ions in our apparatus appear diamond-shaped due to aberrations of unknown origin. We find that as an ion is moved through the focal plane, the width of the point-spread function changes. Consequently, we use this as a substitute for the ion position in the out-of-plane direction.

As an example, fig. S2 shows a stray-field measurement at position $(0, 75\ \mu\mathrm{m}, 0)$. The position and width of the point-spread function on the camera sensor are measured by fitting a contour to it using the OpenCV library (*79*), finding its center and extent. We apply trapping potentials with axial frequencies $\omega_z/2\pi$ of 1.6 MHz or 2.5 MHz, while varying the correction fields in the x, y and z directions. At some correction field $\mathbf{E}_{\mathrm{cross}}$, a crossover point is observed where the ion position or shape is independent of the trapping potential strength. This signifies that this ion position corresponds to the true field null of the applied potential. Linear fits to the data yield $\mathbf{E}_{\mathrm{cross}}$ and an error estimate. Since camera readings can only be specified in terms of integer pixels, error estimates for the x and z-position are ± 0.5 pixels. For the out-of-plane direction, the error on the

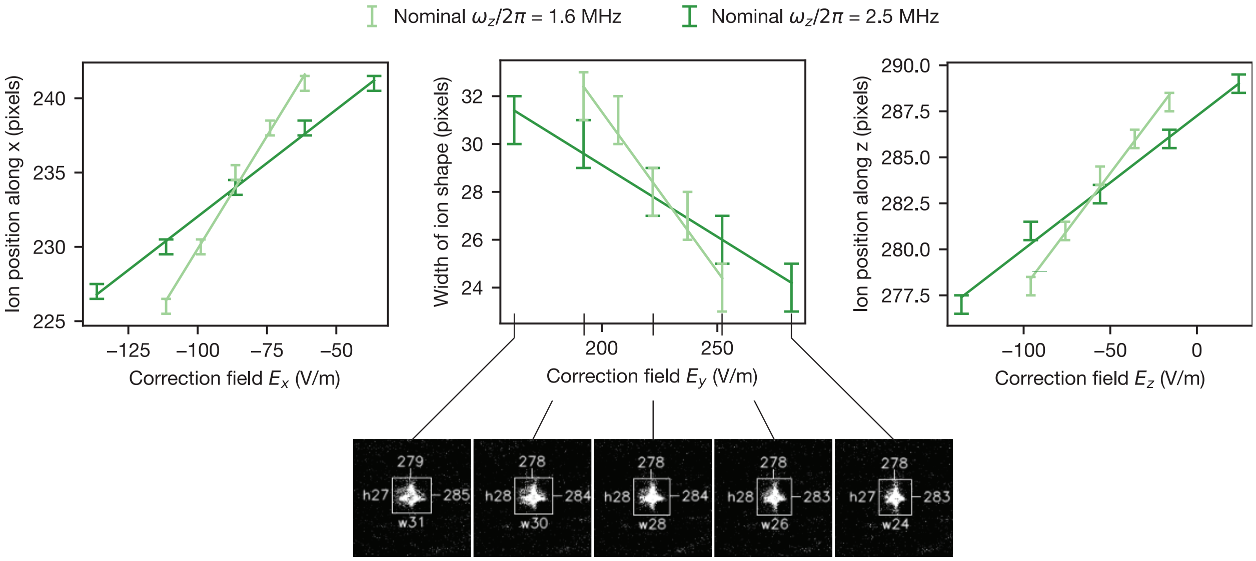

Figure S2: **Measurement and error estimate of the stray field at** $(0, 75\ \mu\mathrm{m}, 0)$**.** Position and width of the ion image on the camera sensor depending on applied correction fields. Light green indicates a nominal axial frequency of the applied potential of 1.6 MHz, while 2.5 MHz was used for data presented in dark green. Error bars are ±0.5 pixels for ion positions along x and z. The middle panel shows the width of the ion image as the ion is moved through the focal plane by an out-of-plane correction field, where error bars of the width are ±1 pixel. The inset below shows the images obtained on the camera, demonstrating the change in aspect ratio. Solid lines are linear fits to the data. From the intersections between these fit lines, the correction fields are found to be $E_{x,\,\mathrm{cross}} = -86.4 \pm 2.0$, $E_{y,\,\mathrm{cross}} = 230.3 \pm 9.0$ and $E_{z,\,\mathrm{cross}} = -59.7 \pm 6.1$.

width of the point-spread function is set to ±1 pixel due to empirically observed fluctuations of the shape readout. We perform such error estimates at a number of positions at every ion–electrode distance to ensure that the changes in the imaging system needed to bring an ion into focus at every distance is well reflected in the error bars of the stray field measurements.

The sensitivity of this measurement method is better understood by estimating it from the experimental parameters. Given the pixel size p of the camera and the imaging magnification M, the minimal detectable position shift is $\pm p/(2M)$, which is approximately 0.25–0.5 µm in our apparatus, due to a pixel size of 16 µm and magnifications in the range of 15-35. The electric field that would cause such a shift in the axial direction is given by $\Delta E_z = mp\omega_z^2/(2eM)$, yielding an estimate for the smallest resolvable electric field in the axial direction. In the radial x-direction, the measurement is twice as sensitive due to the deconfining radial curvature being half the axially confining curvature, assuming a cylindrically symmetric trapping potential. Propagating the error through eq. S3 yields the uncertainty of the measured stray field. The sensitivity benefits from a low ion mass, low trapping potential strength, small camera pixels and a large magnification.

The measurement of out-of-plane fields relies on tracking de-focusing effects. The achievable precision depends on the depth-of-field of the imaging system, given by λ/NA^2 (*80*) in the limit of large NA. For $\lambda \simeq 313$ nm and an NA of 0.55, we obtain a depth of field of approximately 1 µm.

To understand the impact of stray fields with magnitudes up to $\approx 500\ \mathrm{V\,m^{-1}}$ as found in this work, it is worth noting that the position of a $^9\mathrm{Be}^+$ ion in a cylindrically symmetric trapping potential with an axial frequency of 2.6 MHz shifts by 20 µm in the axial direction and −40 µm in a radial direction if subjected to such a field strength.

Complete dataset of stray field measurements

The full dataset of measured stray fields as presented in fig. 2a can be found in fig. S3. A part of the data was retaken about 4 months later as a check of temporal stability. We find that the out-of-plane component of the stray field increased by less than $40\ \mathrm{V\,m^{-1}}$ at a trap height of 152 µm and by about $100\ \mathrm{V\,m^{-1}}$ at $d = 75$ µm. The fields in the x- and z-directions appear to have remained largely constant despite continuous operation of the apparatus.

Error estimates on the measured static electric fields are obtained from linear fits to the ion position/width as a function of the correction field, similar to the procedure shown in fig. S2,

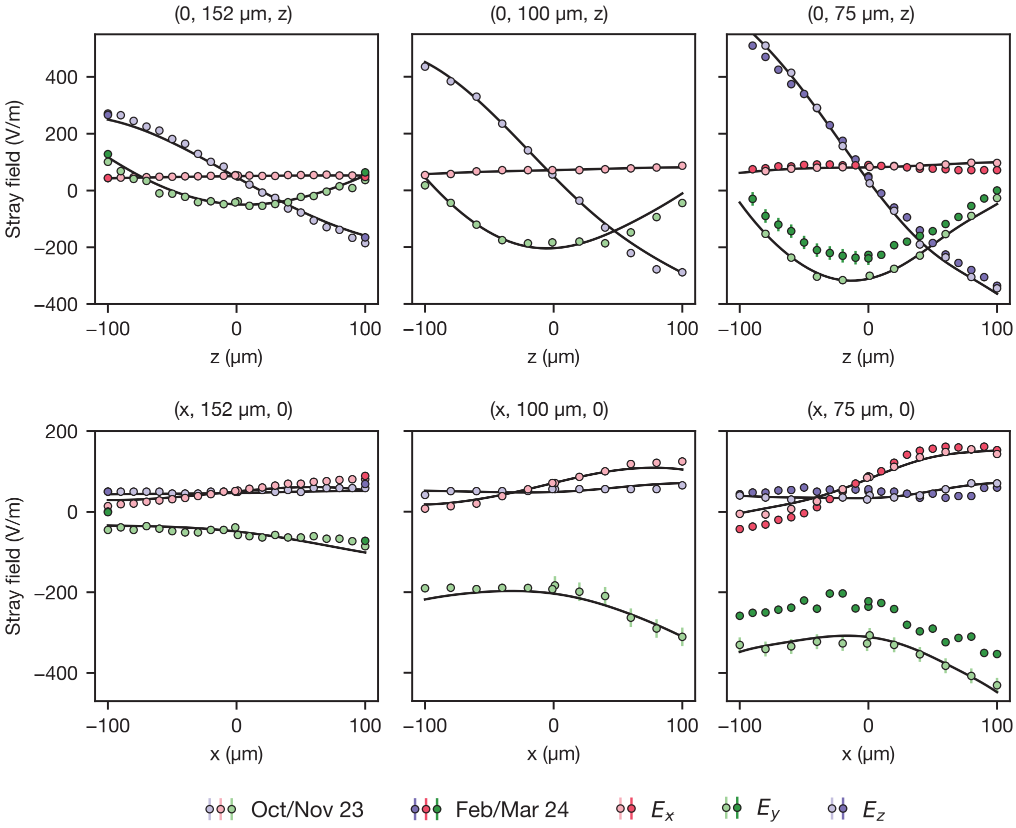

Figure S3: **Detailed stray field data.** Full dataset of measured stray electric fields. The top row depicts fields measured at positions along the axial direction and at three ion-electrode distances, 152 µm, 100 µm, and 75 µm. The bottom row shows fields measured along the radial x-direction. The bulk of the data was taken in October and November 2023 (faded colors) and partially retaken in February and March 2024 (saturated colors) as a check of temporal stability. The data presented in a 3D arrangement in fig. 2a corresponds to the data taken in fall 2023. Solid black lines correspond to the electric fields produced by the fitted dipole density distribution shown in fig. 2b.

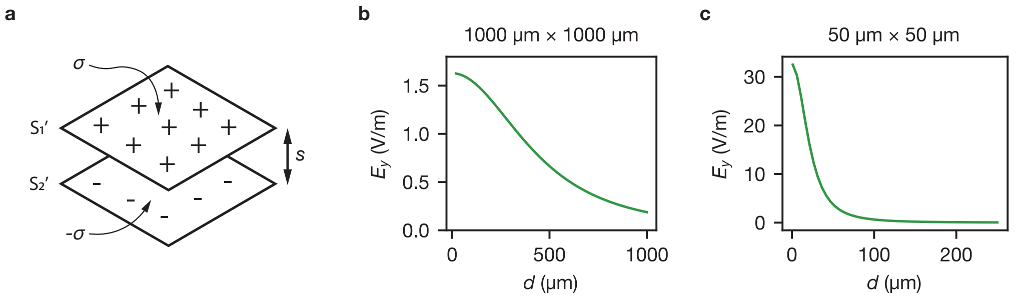

Figure S4: **Dipole patch model. a.** Illustration of a rectangular patch with uniform dipole moment density. Two surfaces with opposite charge density $\pm\sigma$, with a distance s separating them. **b.** The electric fields at a range of distances to the surface d produced by a dipole patch with a dipole moment density $D = 1 \times 10^3\ e\,\text{Å}\,\mu\text{m}^{-2}$ and a size of 1 mm $\times$ 1 mm. **c.** Out-of-plane fields produced by a 50 µm $\times$ 50 µm patch with the same D.

yielding the error of the intercept. We perform such sensitivity scans at a few locations at each trap height used to measure static electric fields. This yields the error estimates for the data shown in fig. S3 and fig. 2a, resulting in estimated standard deviations in the range of 2–6 $\text{V}\,\text{m}^{-1}$ for fields along the z-direction, 1–3 $\text{V}\,\text{m}^{-1}$ for fields in the x-direction, and 10–25 $\text{V}\,\text{m}^{-1}$ for out-of-plane fields.

Simulation of dipole densities on the trap surface

We have measured static electric fields above the trap surface which are not caused by voltages applied externally to the trap electrodes. Such stray electric fields are common in ion traps (*47, 48, 81–85*) and are thought to be caused by several processes, such as laser-induced charging and deposition of neutral atoms on the surface. Assuming that the measured fields indeed originate from charge distributions on the trap surface, we attempt to find their spatial distribution.

Charging through laser light has been investigated in detail (*47, 81*), finding that the resulting electric fields take a dipolar form. It is assumed that the light excites electrons onto insulating patches on the trap surface, forming dipolar fields due to their interaction with the surface. A further mechanism leading to dipole fields is given by adsorbates on the trap surface distorting the electron distributions at the surface (*9, 13, 19, 49, 86, 87*).

We thus model the charge distribution on the trap surface by subdividing it into a number of

small rectangular patches and assuming that each patch is covered in electric dipoles such that the dipole moment density across it is uniform. An illustration of a single patch is given in fig. S4a, showing two surfaces S'_1 and S'_2 separated by s and holding uniform but opposing charge densities $\pm\sigma$. The electrical potential generated by such a charge distribution is

$$\Phi(\mathbf{r}) = \frac{1}{4\pi\epsilon_0}\int_{S'_1}\frac{\sigma}{|\mathbf{r}-\mathbf{r}'|}dS'_1 \tag{S4}$$

$$-\frac{1}{4\pi\epsilon_0}\int_{S'_2}\frac{\sigma}{|\mathbf{r}-\mathbf{r}'|}dS'_2 \, . \tag{S5}$$

We consider the patch to hold a dipole moment density D, where we assume the two surfaces to be infinitesimally close while the charge density grows in such a way that

$$\lim_{s\to 0}(s\cdot\sigma) = D \, . \tag{S6}$$

The potential and electric fields at any position due to a dipole patch can be calculated numerically. As an example, fig. S4b shows the out-of-plane electric field produced by an area of 1 mm × 1 mm covered with a dipole moment density of $D = 1000\ e\,\text{Å}\,\mu\text{m}^{-2}$, while fig. S4c shows the fields produced by a patch of 50 µm × 50 µm with the same value of D. Note that the electric field of a single dipole decays as $1/d^3$ with distance d, whereas fig. S4b and c indicate a slower decay of the field produced by patches of finite size. To find a dipole distribution producing the measured stray fields, we segment a surface area of 1 mm × 1 mm situated at the trap center into 20 × 20 patches, each with an area of 50 µm × 50 µm. By assigning a dipole moment density D_i to the i-th patch, the overall electric field can be calculated. We perform a fit of this model to the data, finding the set of dipole densities $\{D_i\}$, which is plotted in fig. 2b. As this fit is under-constrained (240 measured field values vs. 400 free parameters), we add a regularization term $\sum_i D_i^2$ to the cost function to prevent over-fitting. Due to the resulting fit being biased towards a default state with $D_i = 0$, we additionally allow the entire 1 mm × 1 mm area to hold a background dipole moment density. The fit yields minimal residuals when using $D_{\text{background}} = 180\times10^3\ e\,\text{Å}\,\mu\text{m}^{-2}$. We hypothesize that this background stems from surface dipoles due to adsorbates on the surface. Without knowing the involved species, a realistic estimate for the dipole moment per adsorbed particle is in the range of 1–10 $e\,$Å as found in (*2*). The value of $D_{\text{background}}$ then corresponds to a contaminant density of 18×10^3–$180\times10^3\ \mu\text{m}^{-2}$, well in line with previous investigations (*9*).

Using a fit with patches of different sizes does not considerably alter the resulting dipole distribution, provided that the patches are chosen sufficiently small. Varying the total size of the considered surface area affects the resulting background dipole moment density but leaves the remaining features of the fit intact. Further investigation into the type and spatial distribution of the contaminants would be required to further refine the model. Alternatively, more data at across larger spatial ranges would be necessary.

Motional heating due to electric-field noise

If $r_{\lambda 0}$ is the zero-point rms spread of the wave function, $\boldsymbol{\gamma}_\lambda$ is the normalized mode vector and $\hat{a}_\lambda^\dagger(\hat{a}_\lambda)$ is the creation (annihilation) operator associated with the mode λ, we can write the position vector of the ion as

$$\hat{\mathbf{r}} = -\mathrm{i}\sum_\lambda \sum_\nu r_{\lambda 0}\left[\gamma_{\lambda\nu}^*\hat{a}_\lambda^\dagger - \gamma_{\lambda\nu}\hat{a}_\lambda\right]\mathbf{e}_\nu. \tag{S7}$$

Here the index ν refers to the axes (x, y and z) defined by the coordinate system of the trap such that $\mathbf{e}_\nu$ is the unit vector along that axis. We consider the case when the noisy electric field is along any general direction, so that $\mathbf{E}(t) = E_x(t)\mathbf{e}_x + E_y(t)\mathbf{e}_y + E_z(t)\mathbf{e}_z$. The resulting interaction Hamiltonian is

$$\begin{aligned}\hat{\mathcal{H}}' &= -e\hat{\mathbf{r}}\cdot\mathbf{E}\left(\hat{\mathbf{r}},\omega,t\right)\\ &= \mathrm{i}e\sum_\lambda\sum_\nu r_{\lambda 0}E_\nu\left[\gamma_{\lambda\nu}^*\hat{a}_\lambda^\dagger - \gamma_{\lambda\nu}\hat{a}_\lambda\right].\end{aligned} \tag{S8}$$

Ignoring the spatial variation of the electric field over the motion of the ion and assuming that the noisy field is perturbative compared to the trapping electric field, the heating rate $\dot{n}_\lambda$ of each mode can be derived as (*88*)

$$\dot{n}_\lambda = \frac{e^2 r_{\lambda 0}^2}{2\hbar^2}\sum_\nu \left|\gamma_{\lambda\nu}\right|^2 S_{E_\nu}(\omega_\lambda), \tag{S9}$$

where

$$S_E(\omega) = 4\int_0^\infty \mathrm{d}\tau\langle E_\nu(t')E_\nu(t'+\tau)\rangle \mathrm{e}^{\mathrm{i}\omega\tau} \tag{S10}$$

is the spectral noise density of the electric field along ν.

For a Penning trap with cylindrically symmetric potential, equation S9 simplifies to

$$\dot{n}_z = \frac{e^2}{4\hbar m\omega_z}S_E(\omega_z) \tag{S11}$$

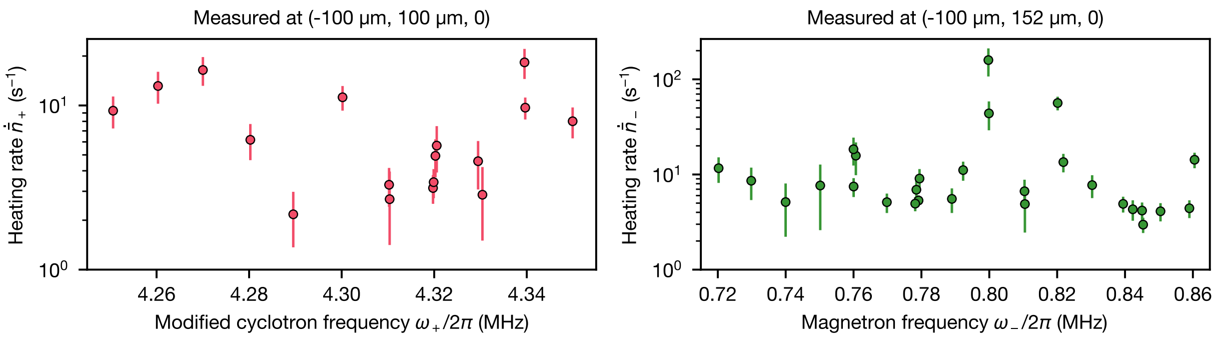

Figure S5: **External noise sources.** Heating rates of the cyclotron and magnetron modes as a function of the mode frequencies, revealing the presence of noise at discrete frequencies. Cyclotron heating rates are measured at a position of $(0, -100\ \mu\text{m}, 100\ \mu\text{m})$, while the ion was placed at $(0, -100\ \mu\text{m}, 152\ \mu\text{m})$ when measuring magnetron heating rates.

for the axial mode, and

$$\dot{n}_\pm = \frac{e^2}{4\hbar m\,(\omega_+ - \omega_-)} S_E(\omega_\pm) \tag{S12}$$

for the radial modes.

Avoiding external noise sources

To ensure comparability, all heating rates presented in this work are taken at the same motional frequencies, except when determining frequency scaling exponents. Axial heating rates are measured at $\omega_z = 2\pi \times 2.6$ MHz, and we ensure the absence of external noise by taking comparison measurements with the electrodes detached from the voltage sources. For the radial modes, we instead perform heating rate measurements across a fine-grained grid of motional frequencies, shown in fig. S5. Noise at discrete frequencies, likely caused by external technical equipment, is detected in this way and can be avoided. For this purpose, we choose to measure all magnetron heating rates at $\omega_- = 2\pi \times 0.845$ MHz and cyclotron heating rates at $\omega_+ = 2\pi \times 4.32$ MHz. Some of the responsible noise sources have been identified, such as a number of optical fiber amplifiers contributing broadband noise above $\simeq 3$ MHz or the EMCCD camera causing discrete noise at a number of frequencies below 1 MHz, while the source of other noise spikes is unknown.

Modeling of the measured electric-field noise

The distance-dependent heating rates shown in fig. 3a exhibit a more complex behavior than the power-law scaling $d^{-\beta}$ expected from microscopic processes on the trap surface. Furthermore, we have shown in appendix 3.4 that the radial modes are affected by external noise sources at discrete frequencies, posing the question if also broadband noise is present. We thus consider that the measured heating rates are caused by multiple noise sources, causing a deviation from the expected scaling laws of surface noise. This hypothesis is further corroborated by the change in frequency-scaling depending on position presented in fig. 3b.

Thermal noise

One source of noise which is guaranteed to be present is Johnson noise (*6*). This type of noise arises due to thermal fluctuations of charge carriers in resistive constituents of the circuitry attached to a trap electrode, such as resistors in filter networks or the bulk electrode material and wiring. We calculate its expected magnitude depending on position and frequency and infer the resulting heating rates of all motional modes. The spectral density of the voltage noise at electrode i produced by this process is given by

$$S_{\mathrm{V},i}(\omega, T) = 4k_{\mathrm{B}}T \cdot \mathrm{Re}\left(Z_i(\omega, T)\right) \ , \tag{S13}$$

where k_{B} is the Boltzmann constant and Z_i is the complex impedance seen by the electrode at a frequency ω and temperature T. Following (*89*), $S_{\mathrm{V},i}$ is converted to an electric-field noise spectral density $S_{\mathrm{E}_\nu,i}$ along the spatial direction ν and at the location $\mathbf{r_0}$. To find this conversion, we calculate the electric field $E_{\nu,i}(\mathbf{r_0})$ generated when applying a voltage V_i to electrode i and define a characteristic distance $d_{\nu,i}$ through

$$E_{\nu,i}(\mathbf{r_0}) = \frac{V_i}{d_{\nu,i}(\mathbf{r_0})} \ . \tag{S14}$$

The spectral density at $\mathbf{r_0}$ is then found by writing

$$S_{E_\nu,i}(\omega, T, \mathbf{r_0}) = \frac{S_{V,i}(\omega, T)}{(d_{\nu,i}(\mathbf{r_0}))^2} \ . \tag{S15}$$

In our apparatus, the major contribution to the impedance causing Johnson noise is due to the last stage of filters placed on a PCB directly below the trap chip, held at a temperature of 6.5 K. Each

electrode is equipped with a resistor-capacitor circuit, with the strongest filtering applied to the five strip electrodes closest to the ion, using $R = 1$ kΩ and $C = 22$ nF. The axially segmented control electrodes are equipped with filters consisting of $R = 10$ kΩ and $C = 560$ pF. The axialization electrodes are equipped with the weakest filtering using a 1 kΩ/560 pF stage, with the electrodes additionally co-wired after the filters. We include resistive elements within the connection between the filters and the trap electrodes, estimated to be no more than 0.25 Ω.

The noise spectral density in both the axial and radial direction is converted to heating rates $\dot{n}_{\lambda,\,\mathrm{Js}}$ where $\lambda \in \{z, +, -\}$ denotes the motional mode. Fig. S6 shows the contributions to the total measured noise. Some striking features caused by the filter network and the electrode geometry are visible. Since only the axially segmented electrodes produce a non-negligible axial electric field, the axial Johnson noise decreases close to the surface. Furthermore, a local minimum in the radial Johnson noise is visible around $d = 152$ µm due to the two axialization electrodes being co-wired and exhibiting an electric-field null.

Correlated technical noise

Motivated by the evidence of the radial modes being affected by external noise (see fig. S5), we model technical noise reaching the trap through electrical connections. Noise originating from the individual digital-to-analog converter (DAC) channels is often considered as a source (*55*). However, DAC-noise is filtered by the cryogenic filters described above as well as fourth-order Butterworth filters (10 kHz cutoff) inserted between the DAC and the vacuum apparatus. The resulting noise suppression is so large that such uncorrelated DAC noise is an unlikely cause of the measured heating rates.

Instead, we consider voltage fluctuations which are correlated across all electrodes. Such noise may arise due to electromagnetic pickup in the trap wiring or due to fluctuations between ground levels in the apparatus. We assume that voltage noise with a spectral density $S_{V,\mathrm{corr}}$ impinges equally on each wire leading to the trap, such that the noise passes through the final stage of cryogenic filters. We further assume for simplicity that the noise is in-phase across all wires and remains so after the filters.

The noise spectral density experienced by the ion in this situation is found by propagating the voltage noise through the trap filters. We find the electric field $E_{\mathrm{corr}}(\omega, \mathbf{r_0})$ experienced by an ion at position $\mathbf{r_0}$ when the set of voltages $V_i = V \cdot |T_i(\omega)|$ is applied to the electrodes. V is the amplitude

of a noise voltage common to all wires and T_i denotes the transfer function of the trap filter of electrode i. The resulting characteristic distance

$$d_{\nu,i}(\omega, \mathbf{r_0}) = \frac{V}{E_{\mathrm{corr},\nu}(\omega, \mathbf{r_0})} \tag{S16}$$

leads to the electric-field spectral density along $\nu \in \{x, y, z\}$

$$S_{E_\nu,\mathrm{corr}}(\omega, \mathbf{r_0}) = \frac{S_{V,\mathrm{corr}}}{(d_{\nu,i}(\omega, \mathbf{r_0}))^2} \ . \tag{S17}$$

Note that this simple model of correlated noise can only explain radial noise. Due to the topology of the filter network, the symmetry of the trap and the assumption of in-phase noise, such fluctuations cannot produce axially polarized noise.

Overall models

We attempt to fit the radial heating rates of mode $\lambda \in \{+, -\}$ with the function

$$\dot{\bar{n}}_\lambda(d) = \dot{\bar{n}}_{\lambda,\,\mathrm{Js}}(d) + C_\lambda \cdot d^{-\beta} + \dot{\bar{n}}_{\lambda,\,\mathrm{corr.}}(d) \ , \tag{S18}$$

which includes Johnson noise and a term with power-law scaling of order β meant to capture surface noise. Finally, we add a contribution $\dot{\bar{n}}_{\lambda,\,\mathrm{corr.}}(d)$ caused by correlated noise with voltage spectral density $S_{V,\mathrm{corr}}$. The free parameters of this model are C_λ, β and $S_{V,\mathrm{corr}}$. The resulting fits to the cyclotron and magnetron data are shown in fig. S6b and c.

Focusing on the fit of the cyclotron heating rates, we observe that correlated technical noise is dominant for most of the considered range of distances, while Johnson noise contributes negligibly. Our model of correlated noise predicts no effect on the ion at a distance slightly above $d = 152$ µm. Similar to the locally minimal level of Johnson noise at $d = 152$ µm, this feature is caused by the fact that the two axialization electrodes are equipped with the weakest filters and thus carry the majority of the noise, leading to an effect similar to an electric-field null of two strip electrodes. The fit favors a power-law noise component with exponent $\beta = 3.5 \pm 0.6$, consistent with the theoretical expectation of $\beta = 4$ and experimental evidence of noise above metallic surfaces. The magnetron mode is less afflicted by correlated noise, similar in magnitude to the expected Johnson noise. Surface noise appears to be the largest contribution to the fit for $d < 152$ µm. Note that the inferred

correlated voltage fluctuations are at a level around 1×10^{-18} V^2 Hz^{-1}, comparable to the noise pickup of a loop of wire with 10 cm diameter in an unshielded indoors environment (*7*).

Correlated technical noise cannot generate axially polarized noise due to the assumption of all noise being in-phase across electrodes, as well as due to the symmetry of the trap and filter network. However, the measured axial heating rates deviate from power-law behavior and approach a constant value at increasing distances to the surface. When modeling the axial heating rates, we thus use the fit function

$$\dot{\bar{n}}_z(d) = \dot{\bar{n}}_{z,\,\mathrm{Js}}(d) + C_z \cdot d^{-\beta} + \dot{\bar{n}}_{z,\,\mathrm{EMI}}\ , \tag{S19}$$

consisting of a term due to Johnson noise, surface noise with power-law distance scaling and a location-independent contribution $\dot{\bar{n}}_{z,\,\mathrm{EMI}}$. A noise source causing this last term could be direct electromagnetic interference (EMI) from the environment of the apparatus (*7*). Fitting this model to the data yields a good fit when assuming EMI noise at a level of $(1.1 \pm 0.2) \times 10^{-16}$ V^2 m^{-2} Hz^{-1}, with the result shown in fig. S6a. Noise levels in an indoors environment can be six orders of magnitude higher (*7*), however detailed simulations would be necessary to find the shielding factor of the experimental apparatus. At distances $d < 152$ µm, noise with power-law scaling dominates the data and we extract $\beta = 4.0 \pm 0.2$, again consistent with expectations.

Finally, we evaluate the obtained noise models on the 3-d grid of positions used in fig. 3d. The resulting heating rates are shown in fig. S6d, where we have used the models with the same fit parameters found for the fits to the distance-dependent data shown in fig. S6a-c. A comparison to the measured values as shown in fig. 3d reveals that the models broadly recover the observed structure for the cyclotron and magnetron data, namely an increase in noise when displaced in either direction along the x-axis. This can be attributed to the reduced distance to the weakly filtered axialization electrodes. We take this agreement as further indication that technical noise of correlated nature is indeed present.

Complete dataset of magnetic field measurements

The full set of magnetic-field gradient data as presented in fig. 4c is shown in fig. S7. Both the shift in the carrier resonance frequency $\Delta\omega$ and the corresponding magnetic-field shift ΔB are shown depending on position, relative to the values measured at position $(0, 152\ \mu\mathrm{m}, 0)$. The displacement

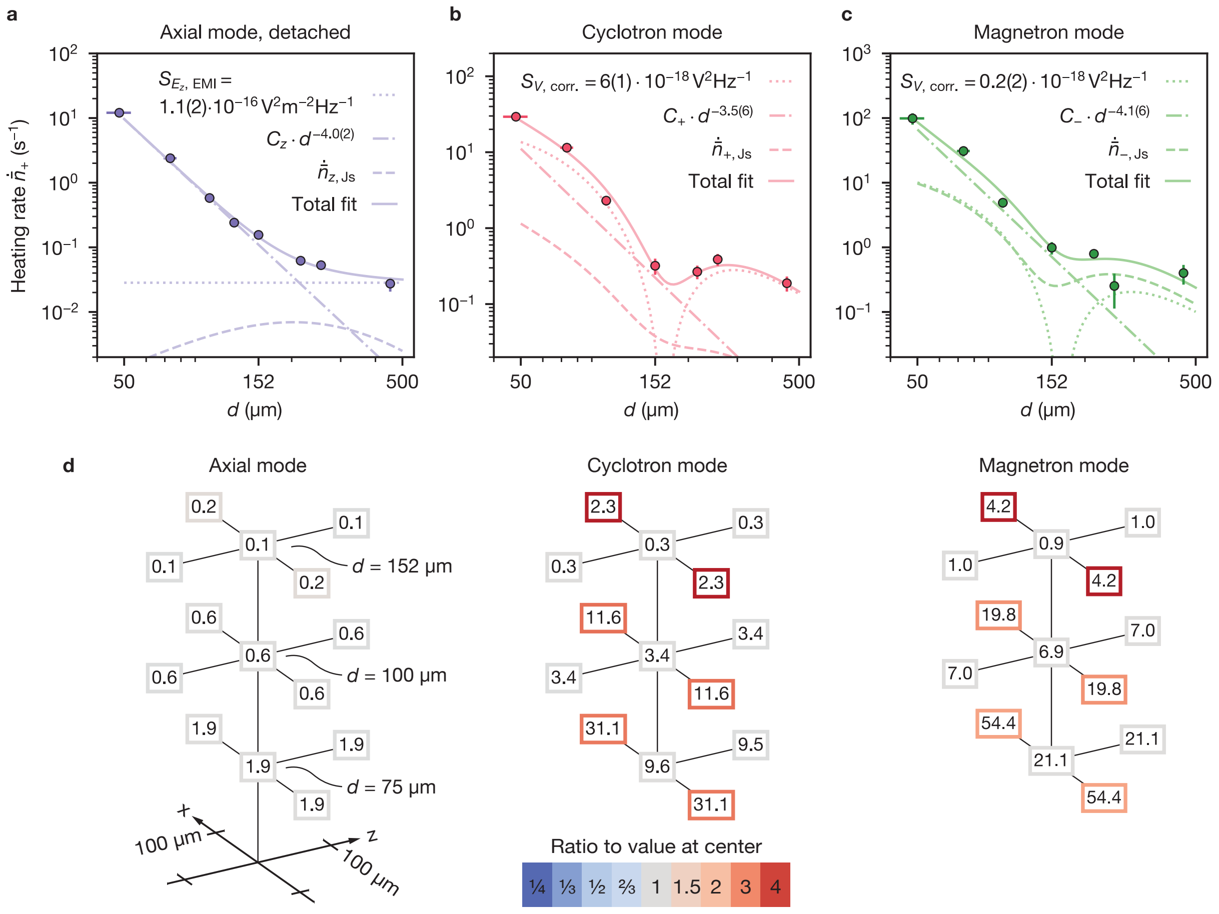

Figure S6: **Models of the measured heating rates. a.** Heating rates of the axial mode taken with the trap electrodes detached, depending on the ion–electrode distance d as presented in fig. 3a. Overlaid is the fit of the model given in eq. S17, including the contributions by the individual sources of noise as described in the text. From the fit component with power-law scaling, assumed to be surface noise, a scaling exponent $\beta = 4.0 \pm 0.2$ is extracted. **b.** Distance-dependent heating rates of the cyclotron mode of motion as shown in fig. 3a and fit of the model given in eq. S18. Correlated voltage fluctuations of $6 \pm 1 \times 10^{-18}$ V^2 Hz^{-1} at the cyclotron frequency of 4.32 MHz are inferred to be impinging on the connections to the trap electrodes. The scaling exponent of the surface noise component is extracted to be $\beta = 3.5 \pm 0.6$. **c.** Magnetron heating rates as given in fig. 3a and fit of the model given in eq. S18. Correlated voltage noise of $0.2 \pm 0.2 \times 10^{-18}$ V^2 Hz^{-1} at the magnetron frequency of 0.845 MHz is inferred from the fit, as well as surface noise with a scaling exponent $\beta = 4.1 \pm 0.6$. **d.** Evaluation of the three fitted heating rate models on the grid of positions used for the measurement of heating rates in 3D presented in fig. 3d, plotted in the same style.

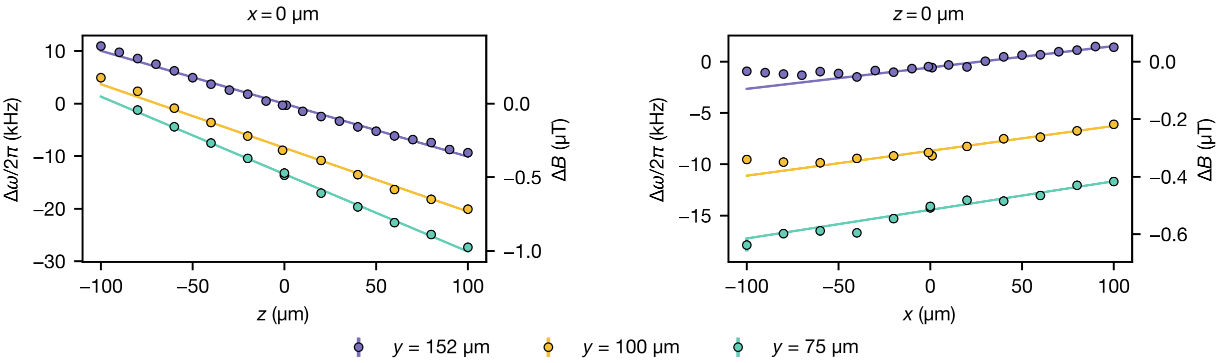

Figure S7: **Detailed measurements of the static magnetic field.** Full dataset of measured magnetic field shifts as a function of location. The left-hand panel shows data taken at locations $(0, y, z)$ for three ion-electrode distances y and with the axial position z ranging from −100–100 μm. Shifts of the resonance frequency are relative to the measured carrier frequency at position $(0, 152\ \mu\text{m}, 0)$. The right-hand panel shows data taken at locations $(x, y, 0)$. Almost all error bars are smaller than the markers. Solid lines indicate linear fits to the data, yielding the magnetic field gradients as detailed in fig. 4.

effects of stray electric fields has been corrected throughout these measurements. Fig. S8 shows the Rabi rates when driving the qubit transition with the microwave field near 83.2 GHz, as presented in fig. 4b and d.

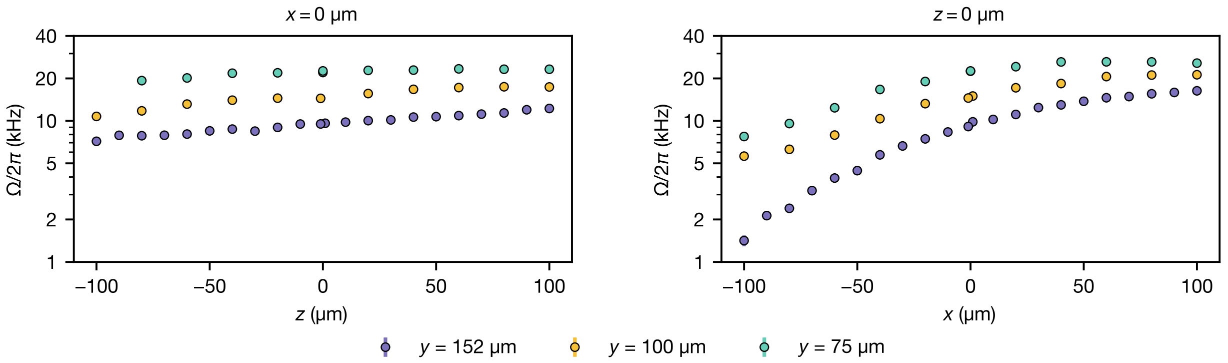

Figure S8: **Detailed microwave Rabi rate measurements.** Full dataset of the measured Rabi rates as a function of location. The left-hand panel shows data taken at locations $(0, y, z)$ for three ion-electrode distances y and with the axial position z ranging from −100–100 µm. The right-hand panel shows data taken at locations $(x, y, 0)$ for the same choices of d. Almost all error bars are smaller than the markers.

REFERENCES

1. N. P. de Leon, K. M. Itoh, D. Kim, K. K. Mehta, T. E. Northup, H. Paik, B. S. Palmer, N. Samarth, S. Sangtawesin, D. W. Steuerman, Materials challenges and opportunities for quantum computing hardware. *Science* **372**, eabb2823 (2021).

2. J. M. Obrecht, R. J. Wild, E. A. Cornell, Measuring electric fields from surface contaminants with neutral atoms. *Phys. Rev. A* **75**, 062903 (2007).

3. J. L. Garrett, J. Kim, J. N. Munday, Measuring the effect of electrostatic patch potentials in casimir force experiments. *Phys. Rev. Res.* **2**, 023355 (2020).

4. F. Antonucci, A. Cavalleri, R. Dolesi, M. Hueller, D. Nicolodi, H. B. Tu, S. Vitale, W. J. Weber, Interaction between stray electrostatic fields and a charged free-falling test mass. *Phys. Rev. Lett.* **108**, 181101 (2012).

5. W.-C. Dong, W.-H. Tan, Z.-J. An, H. Huang, L. Zhu, Y.-J. Tan, T.-Y. Long, C.-G. Shao, S.-Q. Yang, Coupling effect of vibrations and residual electrostatic force in short-range gravitational experiments. *Phys. Rev. Appl.* **20**, 054046 (2023).

6. C. Monroe, D. M. Meekhof, B. E. King, S. R. Jefferts, W. M. Itano, D. J. Wineland, P. Gould, Resolved-sideband Raman cooling of a bound atom to the 3D zero-point energy. *Phys. Rev. Lett.* **75**, 4011–4014 (1995).

7. M. Brownnutt, M. Kumph, P. Rabl, R. Blatt, Ion-trap measurements of electric-field noise near surfaces. *Rev. Mod. Phys.* **87**, 1419–1482 (2015).

8. J. D. Carter, J. D. D. Martin, Coherent manipulation of cold Rydberg atoms near the surface of an atom chip. *Phys. Rev. A* **88**, 043429 (2013).

9. D. Davtyan, S. Machluf, M. L. Soudijn, J. B. Naber, N. J. van Druten, H. B. van Linden van den Heuvell, R. J. C. Spreeuw, Controlling stray electric fields on an atom chip for experiments on Rydberg atoms. *Phys. Rev. A* **97**, 023418 (2018).

10. S. E. de Graaf, L. Faoro, L. B. Ioffe, S. Mahashabde, J. J. Burnett, T. Lindström, S. E. Kubatkin, A. V. Danilov, A. Y. Tzalenchuk, Two-level systems in superconducting quantum devices due to trapped quasiparticles. *Sci. Adv.* **6**, eabc5055 (2020).

11. M. Kim, H. J. Mamin, M. H. Sherwood, K. Ohno, D. D. Awschalom, D. Rugar, Decoherence of near-surface nitrogen-vacancy centers due to electric field noise. *Phys. Rev. Lett.* **115**, 087602 (2015).

12. P. L. Ocola, I. Dimitrova, B. Grinkemeyer, E. Guardado-Sanchez, T. Dordević, P. Samutpraphoot, V. Vuletić, M. D. Lukin, Control and entanglement of individual Rydberg atoms near a nanoscale device. *Phys. Rev. Lett.* **132**, 113601 (2024).

13. T. Thiele, S. Filipp, J. A. Agner, H. Schmutz, J. Deiglmayr, M. Stammeier, P. Allmendinger, F. Merkt, A. Wallraff, Manipulating Rydberg atoms close to surfaces at cryogenic temperatures. *Phys. Rev. A* **90**, 013414 (2014).

14. S. Sangtawesin, B. L. Dwyer, S. Srinivasan, J. J. Allred, L. V. H. Rodgers, K. De Greve, A. Stacey, N. Dontschuk, K. M. O'Donnell, D. Hu, D. A. Evans, C. Jaye, D. A. Fischer, M. L. Markham, D. J. Twitchen, H. Park, M. D. Lukin, N. P. de Leon, Origins of diamond surface noise probed by correlating single-spin measurements with surface spectroscopy. *Phys. Rev. X* **9**, 031052 (2019).

15. K.-Y. Lin, G. H. Low, I. L. Chuang, Effects of electrode surface roughness on motional heating of trapped ions. *Phys. Rev. A* **94**, 013418 (2016).

16. C. Müller, J. H. Cole, J. Lisenfeld, Towards understanding two-level-systems in amorphous solids: Insights from quantum circuits. *Rep. Prog. Phys.* **82**, 124501 (2019).

17. E. Kim, A. Safavi-Naini, D. A. Hite, K. S. McKay, D. P. Pappas, P. F. Weck, H. R. Sadeghpour, Electric-field noise from carbon-adatom diffusion on a au(110) surface: First-principles calculations and experiments. *Phys. Rev. A* **95**, 033407 (2017).

18. J. Lisenfeld, A. Bilmes, A. Megrant, R. Barends, J. Kelly, P. Klimov, G. Weiss, J. M. Martinis, A. V. Ustinov, Electric field spectroscopy of material defects in transmon qubits. *npj Quantum Inf.* **5**, 105 (2019).

19. J. M. McGuirk, D. M. Harber, J. M. Obrecht, E. A. Cornell, Alkali-metal adsorbate polarization on conducting and insulating surfaces probed with bose-einstein condensates. *Phys. Rev. A* **69**, 062905 (2004).

20. J. A. Sedlacek, J. Stuart, D. H. Slichter, C. D. Bruzewicz, R. McConnell, J. M. Sage, J. Chiaverini, Evidence for multiple mechanisms underlying surface electric-field noise in ion traps. *Phys. Rev. A* **98**, 063430 (2018).

21. D. A. Hite, Y. Colombe, A. C. Wilson, K. R. Brown, U. Warring, R. Jördens, J. D. Jost, K. S. McKay, D. P. Pappas, D. Leibfried, D. J. Wineland, 100-fold reduction of electric-field noise in an ion trap cleaned with in situ argon-ion-beam bombardment. *Phys. Rev. Lett.* **109**, 103001 (2012).

22. D. A. Hite, K. S. McKay, D. P. Pappas, Surface science motivated by heating of trapped ions from the quantum ground state. *New J. Phys.* **23**, 103028 (2021).

23. M. Mergenthaler, C. Müller, M. Ganzhorn, S. Paredes, P. Müller, G. Salis, V. P. Adiga, M. Brink, M. Sandberg, J. B. Hertzberg, S. Filipp, A. Fuhrer, Effects of surface treatments on flux tunable transmon qubits. *npj Quantum Inf.* **7**, 157 (2021).

24. M. Alghadeer, A. Banerjee, K. Lee, H. Hussein, H. Fariborzi, S. Rao, Mitigating coherent loss in superconducting circuits using molecular self-assembled monolayers. *Sci. Rep.* **14**, 27340 (2024).

25. P. Chrostoski, H. R. Sadeghpour, D. H. Santamore, Electric noise spectra of a near-surface nitrogen-vacancy center in diamond with a protective layer. *Phys. Rev. Appl.* **10**, 064056 (2018).

26. S. Lin, C. Weng, J. Wang, Y. Guo, Y. Yang, J. Zhao, P. Ma, Y. Chen, L. Lou, W. Zhu, G. Wang, Diamond surface electric-field noise detection using shallow nitrogen-vacancy centers. *Phys. Rev. B* **106**, 165406 (2022).

27. Y. Tao, P. Navaretti, R. Hauert, U. Grob, M. Poggio, C. L. Degen, Permanent reduction of dissipation in nanomechanical si resonators by chemical surface protection. *Nanotechnology* **26**, 465501 (2015).

28. J. A. Sedlacek, J. Stuart, W. Loh, R. McConnell, C. D. Bruzewicz, J. M. Sage, J. Chiaverini, Method for determination of technical noise contributions to ion motional heating. *J. Appl. Phys.* **124**, 214904 (2018).

29. A. Berzins, M. Saleh Ziabari, Y. Silani, I. Fescenko, J. T. Damron, J. F. Barry, A. Jarmola, P. Kehayias, B. A. Richards, J. Smits, V. M. Acosta, Impact of microwave phase noise on diamond quantum sensing. *Phys. Rev. Res.* **6**, 043148 (2024).

30. D. Budker, M. Romalis, Optical magnetometry. *Nat. Phys.* **3**, 227–234 (2007).

31. M. Block, B. Kobrin, A. Jarmola, S. Hsieh, C. Zu, N. Figueroa, V. Acosta, J. Minguzzi, J. Maze, D. Budker, N. Yao, Optically enhanced electric field sensing using nitrogen-vacancy ensembles. *Phys. Rev. Appl.* **16**, 024024 (2021).

32. T. Wolf, P. Neumann, K. Nakamura, H. Sumiya, T. Ohshima, J. Isoya, J. Wrachtrup, Subpicotesla diamond magnetometry. *Phys. Rev. X* **5**, 041001 (2015).

33. M. Simmonds, W. Fertig, R. Giffard, Performance of a resonant input squid amplifier system. *IEEE Trans. Magn.* **15**, 478–481 (1979).

34. T. Bagci, A. Simonsen, S. Schmid, L. G. Villanueva, E. Zeuthen, J. Appel, J. M. Taylor, A. Sørensen, K. Usami, A. Schliesser, E. S. Polzik, Optical detection of radio waves through a nanomechanical transducer. *Nature* **507**, 81–85 (2014).

35. E. Marchiori, L. Ceccarelli, N. Rossi, L. Lorenzelli, C. L. Degen, M. Poggio, Nanoscale magnetic field imaging for 2D materials. *Nat. Rev. Phys.* **4**, 49–60 (2022).

36. Z. Qiu, A. Hamo, U. Vool, T. X. Zhou, A. Yacoby, Nanoscale electric field imaging with an ambient scanning quantum sensor microscope. *npj Quantum Inf.* **8**, 107 (2022).

37. T. Ruster, H. Kaufmann, M. A. Luda, V. Kaushal, C. T. Schmiegelow, F. Schmidt-Kaler, U. G. Poschinger, Entanglement-based dc magnetometry with separated ions. *Phys. Rev. X* **7**, 031050 (2017).

38. A. R. Vasquez, C. Mordini, C. Vernière, M. Stadler, M. Malinowski, C. Zhang, D. Kienzler, K. K. Mehta, J. P. Home, Control of an atomic quadrupole transition in a phase-stable standing wave. *Phys. Rev. Lett.* **130**, 133201 (2023).

39. I. Baumgart, J.-M. Cai, A. Retzker, M. B. Plenio, C. Wunderlich, Ultrasensitive magnetometer using a single atom. *Phys. Rev. Lett.* **116**, 240801 (2016).

40. D. Leibfried, M. D. Barrett, T. Schaetz, J. Britton, J. Chiaverini, W. M. Itano, J. D. Jost, C. Langer, D. J. Wineland, Toward heisenberg-limited spectroscopy with multiparticle entangled states. *Science* **304**, 1476–1478 (2004).

41. K. A. Gilmore, M. Affolter, R. J. Lewis-Swan, D. Barberena, E. Jordan, A. M. Rey, J. J. Bollinger, Quantum-enhanced sensing of displacements and electric fields with two-dimensional trapped-ion crystals. *Science* **373**, 673–678 (2021).

42. H. Wu, G. D. Mitts, C. Z. C. Ho, J. A. Rabinowitz, E. R. Hudson, Wideband electric field quantum sensing via motional raman transitions. *Nat. Phys.* **21**, 380–385 (2025).

43. P. Kaufmann, T. F. Gloger, D. Kaufmann, M. Johanning, C. Wunderlich, High-fidelity preservation of quantum information during trapped-ion transport. *Phys. Rev. Lett.* **120**, 010501 (2018).

44. K. R. Brown, J. Chiaverini, J. M. Sage, H. Häffner, Materials challenges for trapped-ion quantum computers. *Nat. Rev. Mater.* **6**, 892–905 (2021).

45. S. Jain, J. Alonso, M. Grau, J. P. Home, Scalable arrays of micro-penning traps for quantum computing and simulation. *Phys. Rev. X* **10**, 031027 (2020).

46. S. Jain, T. Sägesser, P. Hrmo, C. Torkzaban, M. Stadler, R. Oswald, C. Axline, A. Bautista-Salvador, C. Ospelkaus, D. Kienzler, J. Home, Penning micro-trap for quantum computing. *Nature* **627**, 510–514 (2024).

47. M. Harlander, M. Brownnutt, W. Hänsel, R. Blatt, Trapped-ion probing of light-induced charging effects on dielectrics. *New J. Phys.* **12**, 093035 (2010).

48. S. Auchter, C. Axline, C. Decaroli, M. Valentini, L. Purwin, R. Oswald, R. Matt, E. Aschauer, Y. Colombe, P. Holz, T. Monz, R. Blatt, P. Schindler, C. Rössler, J. Home, Industrially microfabricated ion trap with 1 eV trap depth. *Quantum Sci. Technol.* **7**, 035015 (2022).

49. T. C. Leung, C. L. Kao, W. S. Su, Y. J. Feng, C. T. Chan, Relationship between surface dipole, work function and charge transfer: Some exceptions to an established rule. *Phys. Rev. B* **68**, 195408 (2003).

50. K. Bian, W. Zheng, X. Zeng, X. Chen, R. Stöhr, A. Denisenko, S. Yang, J. Wrachtrup, Y. Jiang, Nanoscale electric-field imaging based on a quantum sensor and its charge-state control under ambient condition. *Nat. Commun.* **12**, 2457 (2021).

51. C. Wagner, M. F. B. Green, M. Maiworm, P. Leinen, T. Esat, N. Ferri, N. Friedrich, R. Findeisen, A. Tkatchenko, R. Temirov, F. S. Tautz, Quantitative imaging of electric surface potentials with single-atom sensitivity. *Nat. Mater.* **18**, 853–859 (2019).

52. S. Zhang, K. Bian, Y. Jiang, Perspective: Nanoscale electric sensing and imaging based on quantum sensors. *Quantum Front.* **2**, 19 (2023).

53. U. Warring, C. Ospelkaus, Y. Colombe, K. R. Brown, J. M. Amini, M. Carsjens, D. Leibfried, D. J. Wineland, Techniques for microwave near-field quantum control of trapped ions. *Phys. Rev. A* **87**, 013437 (2013).

54. F. R. Ong, K. Schüppert, P. Jobez, M. Teller, B. Ames, D. A. Fioretto, K. Friebe, M. Lee, Y. Colombe, R. Blatt, T. E. Northup, Probing surface charge densities on optical fibers with a trapped ion. *New J. Phys.* **22**, 063018 (2020).

55. D. Wineland, C. Monroe, W. Itano, D. Leibfried, B. King, D. Meekhof, Experimental issues in coherent quantum-state manipulation of trapped atomic ions. *J. Res. Natl. Inst. Stand. Technol.* **103**, 259–328 (1998).

56. G. H. Low, P. F. Herskind, I. L. Chuang, Finite-geometry models of electric field noise from patch potentials in ion traps. *Phys. Rev. A* **84**, 053425 (2011).

57. M. Kumph, P. Holz, K. Langer, M. Meraner, M. Niedermayr, M. Brownnutt, R. Blatt, Operation of a planar-electrode ion-trap array with adjustable rf electrodes. *New J. Phys.* **18**, 023047 (2016).

58. L. Martinetz, K. Hornberger, B. A. Stickler, Surface-induced decoherence and heating of charged particles. *PRX Quantum* **3**, 030327 (2022).

59. A. Safavi-Naini, P. Rabl, P. F. Weck, H. R. Sadeghpour, Microscopic model of electric-field-noise heating in ion traps. *Phys. Rev. A* **84**, 023412 (2011).

60. B. L. Foulon, K. G. Ray, C.-E. Kim, Y. Liu, B. M. Rubenstein, V. Lordi, 1/ωelectric-field noise in surface ion traps from correlated adsorbate dynamics. *Phys. Rev. A* **105**, 013107 (2022).

61. C. Noel, M. Berlin-Udi, C. Matthiesen, J. Yu, Y. Zhou, V. Lordi, H. Häffner, Electric-field noise from thermally activated fluctuators in a surface ion trap. *Phys. Rev. A* **99**, 063427 (2019).

62. S. A. King, L. J. Spieß, P. Micke, A. Wilzewski, T. Leopold, J. R. Crespo López-Urrutia, P. O. Schmidt, Algorithmic ground-state cooling of weakly coupled oscillators using quantum logic. *Phys. Rev. X* **11**, 041049 (2021).

63. I. A. Boldin, A. Kraft, C. Wunderlich, Measuring anomalous heating in a planar ion trap with variable ion-surface separation. *Phys. Rev. Lett.* **120**, 023201 (2018).

64. J. A. Sedlacek, A. Greene, J. Stuart, R. McConnell, C. D. Bruzewicz, J. M. Sage, J. Chiaverini, Distance scaling of electric-field noise in a surface-electrode ion trap. *Phys. Rev. A* **97**, 020302 (2018).

65. D. An, C. Matthiesen, E. Urban, H. Häffner, Distance scaling and polarization of electric-field noise in a surface ion trap. *Phys. Rev. A* **100**, 063405 (2019).

66. K. S. McKay, D. A. Hite, P. D. Kent, S. Kotler, D. Leibfried, D. H. Slichter, A. C. Wilson, D. P. Pappas, Measurement of electric-field noise from interchangeable samples with a trapped-ion sensor. *Phys. Rev. A* **104**, 052610 (2021).

67. M. Héritier, R. Pachlatko, Y. Tao, J. M. Abendroth, C. L. Degen, A. Eichler, Spatial correlation between fluctuating and static fields over metal and dielectric substrates. *Phys. Rev. Lett.* **127**, 216101 (2021).

68. N. Shiga, W. M. Itano, J. J. Bollinger, Diamagnetic correction to the $^{9}Be^{+}$ ground-state hyperfine constant. *Phys. Rev. A* **84**, 012510 (2011).

69. C. L. Degen, F. Reinhard, P. Cappellaro, Quantum sensing. *Rev. Mod. Phys.* **89**, 035002 (2017).

70. T. Ruster, C. T. Schmiegelow, H. Kaufmann, C. Warschburger, F. Schmidt-Kaler, U. G. Poschinger, A long-lived zeeman trapped-ion qubit. *Appl. Phys. B* **122**, 254 (2016).

71. H. Q. Fan, S. Kumar, R. Daschner, H. Kübler, J. P. Shaffer, Subwavelength microwave electric-field imaging using Rydberg atoms inside atomic vapor cells. *Opt. Lett.* **39**, 3030–3033 (2014).

72. M. A. Weber, C. Löschnauer, J. Wolf, M. F. Gely, R. K. Hanley, J. F. Goodwin, C. J. Ballance, T. P. Harty, D. M. Lucas, Cryogenic ion trap system for high-fidelity near-field microwave-driven quantum logic. *Quantum Sci. Technol.* **9**, 015007 (2024).

73. S. C. Burd, R. Srinivas, J. J. Bollinger, A. C. Wilson, D. J. Wineland, D. Leibfried, D. H. Slichter, D. T. C. Allcock, Quantum amplification of mechanical oscillator motion. *Science* **364**, 1163–1165 (2019).

74. A. R. Milne, C. Hempel, L. Li, C. L. Edmunds, H. J. Slatyer, H. Ball, M. R. Hush, M. J. Biercuk, Quantum oscillator noise spectroscopy via displaced cat states. *Phys. Rev. Lett.* **126**, 250506 (2021).

75. J. C. Thomas, J. J. Schwartz, J. N. Hohman, S. A. Claridge, H. S. Auluck, A. C. Serino, A. M. Spokoyny, G. Tran, K. F. Kelly, C. A. Mirkin, J. Gilles, S. J. Osher, P. S. Weiss, Defect-

tolerant aligned dipoles within two-dimensional plastic lattices. *ACS Nano* **9**, 4734–4742 (2015).

76. R. Lu, A. J. Elliot, L. Wille, B. Mao, S. Han, J. Z. Wu, J. Talvacchio, H. M. Schulze, R. M. Lewis, D. J. Ewing, H. F. Yu, G. M. Xue, S. P. Zhao, Fabrication of Nb/Al_2O_3/Nb josephson junctions using in situ magnetron sputtering and atomic layer deposition. *IEEE Trans. Appl. Supercond.* **23**, 1100705–1100705 (2013).

77. R. Kumar, S. Mahajan, F. Donaldson, S. Dhomkar, H. J. Lancaster, C. Kalha, A. A. Riaz, Y. Zhu, C. A. Howard, A. Regoutz, J. J. L. Morton, Stability of Near-Surface nitrogen vacancy centers using dielectric surface passivation. *ACS Photonics* **11**, 1244–1251 (2024).

78. C. Mordini, F. Lancellotti, V. Negnevitsky, M. Marinelli, R. Oswald, T. Saegesser, pytrans (v2.1.0+a). Zenodo (2023); https://doi.org/10.5281/zenodo.10204606.

79. G. Bradski, The OpenCV Library. *Dr. Dobb's J. Softw. Tools* **25**, 120–126 (2000).

80. D. B. Murphy, M. W. Davidson, "Diffraction and spatial resolution" in *Fundamentals of Light Microscopy and Electronic Imaging* (John Wiley & Sons, Ltd, ed. 2, 2012), chap. 6, p. 109.

81. S. X. Wang, G. Hao Low, N. S. Lachenmyer, Y. Ge, P. F. Herskind, I. L. Chuang, Laser-induced charging of microfabricated ion traps. *J. Appl. Phys.* **110**, 104901 (2011).

82. W. Lee, D. Chung, H. Jeon, B. Cho, K. Choi, S. Yoo, C. Jung, J. Jeong, C. Kim, D.-I. D. Cho, T. Kim, Photoinduced charge-carrier dynamics in a semiconductor-based ion trap investigated via motion-sensitive qubit transitions. *Phys. Rev. A* **109**, 043106 (2024).

83. A. Härter, A. Krükow, A. Brunner, J. Hecker Denschlag, Long-term drifts of stray electric fields in a paul trap. *Appl. Phys. B* **114**, 275–281 (2014).

84. S. C. Doret, J. M. Amini, K. Wright, C. Volin, T. Killian, A. Ozakin, D. Denison, H. Hayden, C.-S. Pai, R. E. Slusher, A. W. Harter, Controlling trapping potentials and stray electric fields in a microfabricated ion trap through design and compensation. *New J. Phys.* **14**, 073012 (2012).

85. S. Narayanan, N. Daniilidis, S. A. Möller, R. Clark, F. Ziesel, K. Singer, F. Schmidt-Kaler, H. Häffner, Electric field compensation and sensing with a single ion in a planar trap. *J. Appl. Phys.* **110**, 114909 (2011).

86. A. Tauschinsky, R. M. T. Thijssen, S. Whitlock, H. B. van Linden van den Heuvell, R. J. C. Spreeuw, Spatially resolved excitation of Rydberg atoms and surface effects on an atom chip. *Phys. Rev. A* **81**, 063411 (2010).

87. J. A. Sedlacek, E. Kim, S. T. Rittenhouse, P. F. Weck, H. R. Sadeghpour, J. P. Shaffer, Electric field cancellation on quartz by Rb adsorbate-induced negative electron affinity. *Phys. Rev. Lett.* **116**, 133201 (2016).

88. T. A. Savard, K. M. O'Hara, J. E. Thomas, Laser-noise-induced heating in far-off resonance optical traps. *Phys. Rev. A* **56**, R1095–R1098 (1997).

89. D. Leibrandt, B. Yurke, R. Slusher, Modeling ion trap thermal noise decoherence. *Quant. Inf. Comput.* **7**, 052–072 (2007).
